# RNA editing of microtubule-associated protein tau circular RNAs promotes their translation and tau tangle formation

**DOI:** 10.1093/nar/gkac1129

**Published:** 2022-12-19

**Authors:** Justin Ralph Welden, Giorgi Margvelani, Karol Andrea Arizaca Maquera, Bhavani Gudlavalleti, Sandra C Miranda Sardón, Alexandre Rosa Campos, Noémie Robil, Daniel C Lee, Alvaro G Hernandez, Wang-Xia Wang, Jing Di, Pierre de la Grange, Peter T Nelson, Stefan Stamm

**Affiliations:** Sanders-Brown Center on Aging, University of Kentucky, Lexington, KY, USA; Department of Molecular and Cellular Biochemistry, University of Kentucky, Lexington, KY, USA; Department of Molecular and Cellular Biochemistry, University of Kentucky, Lexington, KY, USA; Department of Molecular and Cellular Biochemistry, University of Kentucky, Lexington, KY, USA; Department of Molecular and Cellular Biochemistry, University of Kentucky, Lexington, KY, USA; Sanford Burnham Prebys Medical Discovery Institute Proteomics Core, La Jolla, CA, USA; GenoSplice, Paris, France; Sanders-Brown Center on Aging, University of Kentucky, Lexington, KY, USA; Alzheimer's Disease Research Center Neuroscience, University of Kentucky, Lexington, KY, USA; DNA Services Facility, University of Illinois, Urbana, IL, USA; Sanders-Brown Center on Aging, University of Kentucky, Lexington, KY, USA; Alzheimer's Disease Research Center and Department of Pathology and Laboratory Medicine, University of Kentucky, Lexington, KY, USA; Alzheimer's Disease Research Center and Department of Pathology and Laboratory Medicine, University of Kentucky, Lexington, KY, USA; GenoSplice, Paris, France; Sanders-Brown Center on Aging, University of Kentucky, Lexington, KY, USA; Alzheimer's Disease Research Center and Department of Pathology and Laboratory Medicine, University of Kentucky, Lexington, KY, USA; Department of Molecular and Cellular Biochemistry, University of Kentucky, Lexington, KY, USA

## Abstract

Aggregation of the microtubule-associated protein tau characterizes tauopathies, including Alzheimer's disease and frontotemporal lobar degeneration (FTLD-Tau). Gene expression regulation of tau is complex and incompletely understood. Here we report that the human tau gene (*MAPT*) generates two circular RNAs (circRNAs) through backsplicing of exon 12 to either exon 7 (12→7 circRNA) or exon 10 (12→10 circRNA). Both circRNAs lack stop codons. The 12→7 circRNA contains one start codon and is translated in a rolling circle, generating a protein consisting of multimers of the microtubule-binding repeats R1–R4. For the 12→10 circRNA, a start codon can be introduced by two FTLD-Tau mutations, generating a protein consisting of multimers of the microtubule-binding repeats R2–R4, suggesting that mutations causing FTLD may act in part through tau circRNAs. Adenosine to inosine RNA editing dramatically increases translation of circRNAs and, in the 12→10 circRNA, RNA editing generates a translational start codon by changing AUA to AUI. Circular tau proteins self-aggregate and promote aggregation of linear tau proteins. Our data indicate that adenosine to inosine RNA editing initiates translation of human circular tau RNAs, which may contribute to tauopathies.

## INTRODUCTION

Under pathophysiological conditions, the human microtubule protein tau (MAPT) can misfold into intracellular, insoluble protein polymers known as paired helical filaments (PHFs). In turn, PHFs coalesce into neurofibrillary tangles (NFTs) which characterize a group of neurodegenerative diseases known as tauopathies, that include Alzheimer's disease (AD) and frontotemporal lobar degeneration (FTLD-TAU) (1). We refer to the protein as tau, the gene as *MAPT* and the protein made from circular RNA as circ tau protein. The clearest connection between the *MAPT* gene and neurodegeneration is found in FTLD-TAU, as the disease is caused by at least 53 known mutations in *MAPT* (2,3). Clinical–pathological correlation studies in AD indicate that NFTs are the pathological feature most strongly associated with cognitive status (4). Through their microtubule-binding repeats, tau proteins bind to and stabilize microtubules (4,5). These repeat domains are encoded by exons 7–12 of the *MAPT* pre-mRNA that contains at least 16 exons, with exons 2, 3, 4a, 6, 8 and 10 being alternatively spliced cassette exons. The *MAPT* gene encompasses ∼62 kbp and contains at least 83 Alu elements—56 in sense and 27 in antisense orientation (6). Exon 10 encodes the second microtubule-binding repeat and is alternatively spliced in adult human brain that contains a mixture of tau proteins with three or four microtubule-binding repeats (7).

In addition to linear mRNAs, *MAPT* pre-mRNA forms circular RNAs (circRNAs) (8). CircRNAs are expressed at low levels, usually <1% of their linear counterparts (9,10), but collectively are prevalent in the brain (11–14). CircRNAs are made from pre-mRNA transcripts through backsplicing, where a 5′ splice site is not linked to a downstream, but to an upstream 3′ splice site. Recent work shows that circRNAs can be translated (15–19). However, the mechanism leading to translational initiation remains obscure, since circRNAs lack an RNA cap structure and in general have no known ribosomal entry sites.

Due to the circular nature of these RNAs, the translated proteins cannot be fully predicted from the ‘linear’ genome and can acquire novel functions. CircRNAs are more stable than linear mRNAs, are usually located in the cytosol and, in the brain, are enriched in synaptosomes. For formation, most primate circRNAs depend on pre-mRNA structures imposed by primate-specific intronic Alu elements (20–25) that are extensively (>85%) (26,27) modified by ADAR enzymes (adenine deaminase acting on RNA). ADAR enzymes recognize double-stranded RNA structures and convert adenosines into inosines (28). Humans express three ADAR genes, with ADAR1 and 2 catalytically active whereas ADAR3 is catalytically inactive (29).

**Figure 1.**
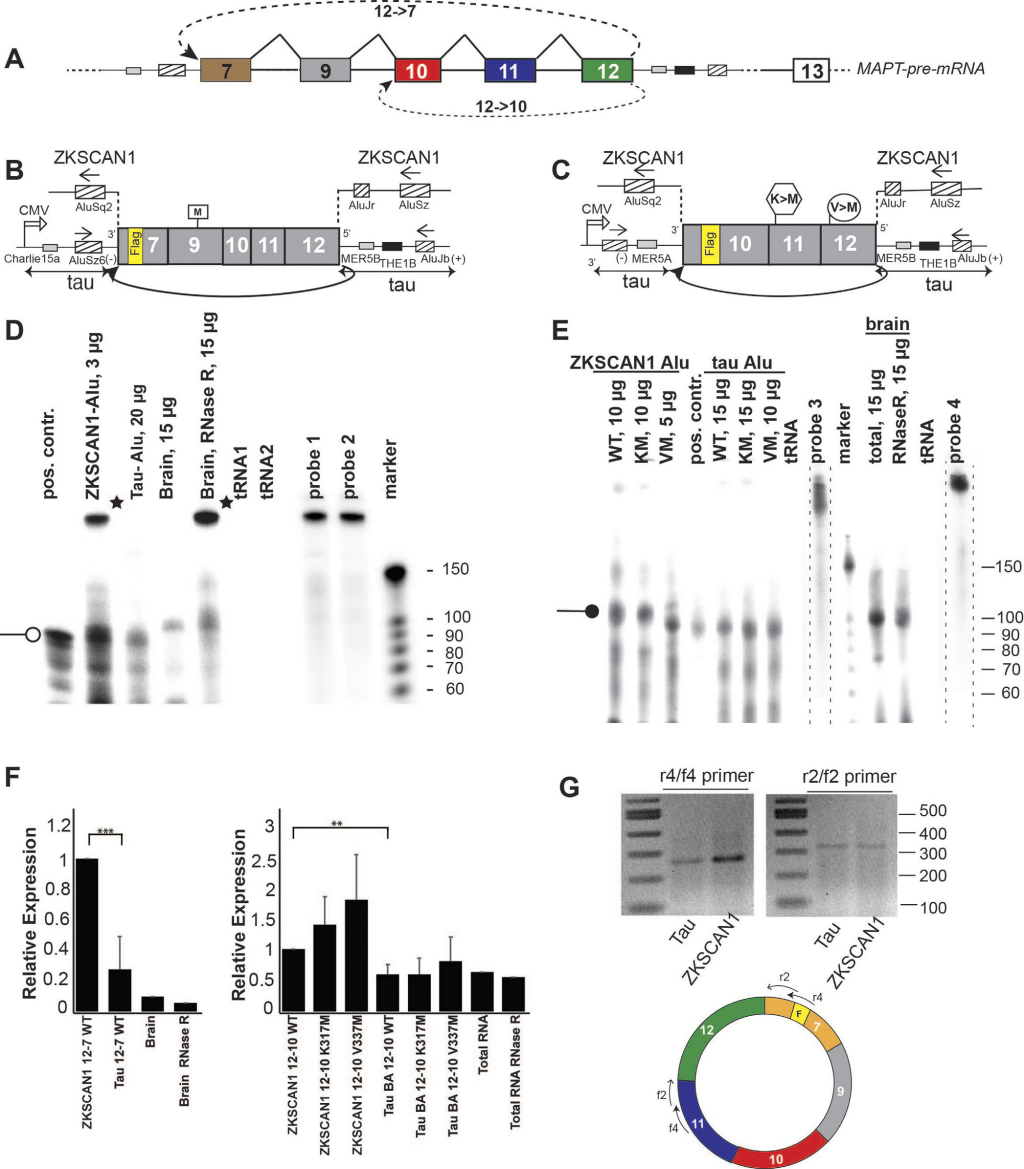
Tau circRNAs 12→7 and 12→10 are expressed in brain and can be expressed from cDNA constructs. (**A**) Schematic representation of the *MAPT* region that undergoes backsplicing to generate the 12→7 and 12→10 tau circRNAs. The intronic parts of intron 6 and 12 included in the expression constructs are schematically indicated. (**B**) Schematic representation of the 12→7 expression constructs. The introns between exons 7 and 12 were removed and a 3× Flag tag was introduced in exon 7. Two variants were constructed: one variant was flanked by the Alu elements of the *ZKSCAN1* gene and a second variant was flanked by the first 1000 nucleotides of *MAPT* introns 6 and 12 that contain two Alu elements in antisense orientation. The coordinates of the exons are given in the Materials and Methods. (**C**) Schematic representation of the 12→10 expression constructs. The introns between exons 10 and 12 were removed and a 3× Flag tag was introduced into exon 10. This sequence is flanked by either *ZKSCAN1* introns or ∼1000 nt of the wild-type *MAPT* introns 9 and 12 that contain two Alu elements in antisense orientation. (**D**) RNase protection assay of RNA made from cells transfected with 12→7 constructs and human entorhinal cortex (Braak stage 0, i.e. no tau pathology) using the amount of total RNA indicated. tRNAs: *E. coli* tRNAs as negative controls. Probe 1 detects RNA from cell lines, probe 2 detects endogenous RNAs. An open round arrowhead indicates the protected fragment. Stars indicate incomplete probe digestion due to contaminating DNA. The total brain RNA is not RNase R treated and compared with RNase R-treated brain RNA. (**E**) RNase protection assay of RNA made from cells transfected with 12→10 constructs and human hippocampus RNA cortex (Braak 0). Probe 3 detects RNA from cell lines, probe 4 detects endogenous RNAs. The probes are from a shorter exposure, indicated by dotted lines. A round arrowhead indicates the protected fragment. The location of the probes is shown in Supplementary Figure S1. The total brain RNA is not RNase R treated and compared with RNase R-treated brain RNA. (**F**) Quantification of three independent experiments, the signals of the 100 nt long protected fragment was normalized to the 12→7 ZKSCAN1 WT (left) and 12→10 ZKSCAN1 WT signal (right), taking the amount of analyzed RNA into account. (**G**) RT–PCR to validate the 12→7 circRNA generated by transfecting the expression construct in HEK293T cells. The cartoon below shows the location of the two RT–PCR primer sets used.

*MAPT* generates circRNAs through backsplicing of its exon 12 to either exon 10 (12→10) or 7 (12→7) (Figure 1A–C; Supplementary Figure S1A, B). The exons 13, 10 and 7 involved in the backsplice site selection are flanked by Alu elements in antisense orientation that in general facilitate the backsplicing (30).

The resulting two tau circRNA species contain a number of nucleotides exactly divisible by three and are lacking an in-frame stop codon. The 12→7 circRNA contains an in-frame start codon, whereas the 12→10 circRNA lacks a start codon. Thus, if translation initiates, these circRNAs could form multimers due to rolling circle translation, where a ribosome translates the circRNA several times (Supplementary Figure S1C, D).

Here we report that tau circRNAs are translated, which is strongly promoted by adenosine to inosine (A>I) editing, catalyzed by ADAR enzymatic activity. The ADAR dependency of translation is also seen for tau circRNAs derived from a group I intron, ruling out *trans*-splicing effects (19). In addition, A>I editing changes the encoded protein, for example by changing an AUA (Ile) to AUI (start) codon, which initiates translation of the start codon-less 12→10 circRNA. Functionally, circ tau proteins promote tau aggregation, indicating that they could contribute to tauopathies.

## MATERIALS AND METHODS

### Cloning and availability of constructs

DNA constructs were made by Gibson Cloning (31) (New England Biolabs, NEB builder). All new constructs were deposited into Addgene. The genomic coordinates in GRCh38/hg38 were: exon 7, chr17_KI270908v1_alt:693 579–693 705; exon 9, chr17_KI270908v1_alt:698 518–698 782; exon 10, chr17_KI270908v1_alt:712 429–712 521; exon 11, chr17_KI270908v1_alt:716 362–716 443; and exon 12, chr17_KI270908v1_alt:720 732–720 844.

### RNase protection

RNase protection was performed as described (32) using uniformly ^32^P-labeled probes [2.5 × 10^6^–3 × 10^6^ counts per minute (cpm), α-UTP 800 Ci/mmol)] in RNase hybridization buffer (80% formamide, 300 mM NaOAc, 100 mM NaCitrate, 1 mM EDTA, 5 % polyethylene glycol 5000) overnight at 55°C. Digestion was performed using 3 μl of RNase A1/T1 in 150 μl of 15 mM NaCl, 10 mM Tris–HCl, pH 7, followed by phenol extraction and precipitation. RNA was isolated using Qiagen RNeasy columns, and RNase R digestion was performed according to the manufacturer's protocol (BioVision RNase R, M1228-500).

### RT–PCR for circular RNAs

Reverse transcription–polymerase chain reaction (RT–PCR) was performed as described (8,33). Primers spanning the 12→7 exon junction were 7_12 circdetect rev4 ACCGTCATGGTCTTTGTAGTC, 7_12 circdetect for4 AATATCAAACACGTCCCGGGA and 7_12 circdetect Fw2: AATAAGAAGCTGGATCTTAGC, 7_12 circdetect rev2: GATCTTTATAATCACCGTCAT

### Transient transfections

Transient transfections were performed in human embryo kidney (HEK) 293T cells. A 4 μg aliquot of plasmid DNA was mixed with 200 μl of sterile 150 mM NaCl in a ratio of 1 μg DNA per 3 μl polyethylenimine solution (1 μg/μl) (PEI; Polysciences, 24765-1). Co-transfections were at a 1:1 ratio (4 μg:4 μg). The DNA was incubated at room temperature for 20 min and then added to the cells. The cells were cultured in a 150 mm dish (Azer Scientific, ES56268), and transfected at 60% confluency. The cells were lysed and analyzed 96 h post-transfection.

### Circ tau protein

The circ tau protein was isolated as described in Supplementary Figure S2 through immunoprecipitation from cellular extracts using 10 μl of M2 anti-Flag magnetic beads per 1 mg of total protein (in S3 lysate). Tau circular protein is heat stable and we could not detect Flag immunoreactivity in the pellets P1–P3 (Supplementary Figure S2).

Protein was eluted using 15 μg of anti-Flag peptide and concentrated using Amicon Ultra 0.5 ml centrifugal filters, Ultracel-3K. In other immunoprecipitation experiments, protein was eluted by boiling in sodium dodecylsulfate (SDS) loading buffer.

Tau fibrils were generated by mixing 2 μg of purified circ tau protein with 1 μg of linear 2N4R tau protein at a 1:1 molar ratio in the presence of 500 ng of yeast RNA (34) and incubating for 3 days.

Biosensor cells were obtained from the ATCC (CRL-3275) and treated with 0.5 μg of pre-formed fibrils with Lipofectamine 2000.

### DNA expression constructs

The DNA expression constructs were made in pcDNA3.1 using Gibson cloning (31). Intronic coordinates were chr17_KI270908v1_alt:692 195–721 801 (Hg38) without introns 10, 11 and 12 for the 12→10 constructs and chr17_KI270908v1_alt:692 195–721 801 (Hg38) without introns 7–12 for the 12→7 constructs. All constructs are available from Addgene.

### CircRNA sequencing

Construction of libraries and sequencing on the Illumina NovaSeq 6000 were performed at the Roy J. Carver Biotechnology Center at the University of Illinois at Urbana-Champaign. rRNA was removed using the Ribo minus kit (Thermo). After purification with RNA cleanup columns (Zymo Research), the RNAs were incubated with RNase R (NEB) at 37°C for 15 min, followed by purification with RNA cleanup columns. Eluted RNAs were converted into RNAseq libraries with the TruSeq Stranded Total RNA kit (Illumina), starting at the fragmentation step. Briefly, the circRNAs were chemically fragmented, annealed with a random hexamer and converted to double-stranded cDNAs, which were subsequently blunt-ended, 3′-end A-tailed and ligated to indexed adaptors. Each library was ligated to a uniquely dual indexed adaptor (unique dual indexes) to prevent index switching. The adaptor-ligated double-stranded cDNAs were amplified by PCR for eight cycles with the Kapa HiFi polymerase (Roche, CA, USA) to reduce the likelihood of multiple identical reads due to preferential amplification. The final libraries were quantitated with Qubit (ThermoFisher, MA, USA) and the average library fragment length was determined on a Fragment Analyzer. The libraries were diluted to 10 nM and further quantitated by quantitative PCR (qPCR) on a CFX Connect Real-Time qPCR system (Biorad, Hercules, CA, USA) for accurate pooling of the barcoded libraries and maximization of the number of clusters in the flowcell.

### RNA-Seq data analysis

Backsplice junctions were searched within raw sequences using zgrep with ATTAATTATCTGCACCTTTTTATTTCCTCC for R1 files or GGAGGAAATAAAAAGGTGCAGATAATTAAT for R2 files. Subsequent read IDs (both reads of each positive pair) were used to define new fastq files using the subseq function from seqtk (v1.0-r32). Reads were then aligned to the reference tau circRNA fasta file (including Tag) using Bowtie2 (v2.2.3) and the ‘–very-sensitive-local’ parameter. Samtools (v1.11) mpileup and pileup2base script were used to study sequence composition at each position. Only positions with A>G and at least 1% of ‘G’ in one sample were considered.

### Transmission electron microscopy (TEM)

Following *in vitro* tau fibril formation, the fibril samples were subjected to sonication for 10 min in a water bath. Prior to sample preparation for TEM, all grids were pre-exposed to UV light for 1 h. A 1 μl aliquot of sonicated fibril sample was loaded onto a carbon-coated, 300 mesh copper grid (Electron Microscopy Sciences, CF300-CU-50) and allowed to be absorbed for 1 min. The excess liquid was then wicked off. Negative staining was carried out by applying 1 μl of 2% uranyl acetate to the grid and incubating for 30 s. The excess stain was wicked off and the grid was subsequently washed twice with a drop of deionized water. The grid was air-dried prior to imaging using a FEI Talos F200X TEM (ThermoFisher Scientific) at the University of Kentucky Electron Microscopy Center.

## RESULTS

### Reporter systems recapitulated the expression of tau circRNAs seen in human brain

To characterize the tau circRNAs, we generated two cellular expression systems. In the first, the cDNA corresponding to exon 12 backspliced to either exon 7 or exon 10 is flanked by the heterologous introns of the *ZKSCAN1* gene. *ZKSCAN1* contains a total of three Alu elements (Figure 1B, C), which flank one of the most highly expressed human circRNAs (35). In the second series of constructs, the *MAPT* cDNAs are flanked by ∼1 kb of their wild-type *MAPT* intron regions (Figure 1A–C). Each of these regions contains an Alu element in antisense orientation relative to the Alu element on the opposite side, which generally promotes backsplicing (30). To distinguish the tau circRNA from endogenous RNA, we introduced a 3× Flag tag in exon 7 or 10, respectively (Figure 1B, C). Our constructs had no internal introns, allowing us to focus on analyzing the single backsplicing event from exon 12 to either exon 10 or exon 7.

These constructs were transfected into HEK293T cells and their RNA expression was determined using RNase protection as described earlier (8), employing probes that cover the 12→10 and 12→7 junctions, respectively (Supplementary Figure S1A, B). For each circRNA, we used a probe for the endogenous RNA (probe 1) and a probe that detects the RNA expressed from the expression constructs that covers the Flag tag (probe 2). The 12→7 cDNA generated a fragment of the expected size, when flanked both with heterologous *ZKSCAN1* introns and with the wild-type *MAPT* introns. Flanking the cDNA with the wild-type *MAPT* introns reduced the expression levels ∼5-fold compared with the *ZKSCAN1* introns (Figure 1D, F).

Next, we tested the expression of the 12→7 circRNA in human brain and analyzed total and RNase R-treated entorhinal cortex RNA. RNase R digests linear RNAs and thus enriches for circRNAs. As shown in Figure 1D, we observed the protected exon junction fragment that is resistant to RNase R treatment in brain, showing the circular nature of the endogenously expressed tau circRNA.

The formation of the 12→10 circRNA from the expression constructs was tested similarly, together with the two frontotemporal lobar degeneration (2) mutations K317M (36) and V337M (37,38) that introduce start codons into the 12→10 tau circRNA (Figure 1C). Again, we saw a higher expression from the constructs surrounded by *ZKSCAN1* introns when compared with constructs surrounded by wild-type *MAPT* introns (Figure 1E). Combining the results of three independent experiments showed that *ZKSCAN1* introns increased the expression levels ∼2-fold. The presence of the FTLD-Tau mutations had no effect on RNA expression from constructs flanked by wild-type *MAPT* introns. The mutations showed a trend to increase the expression level in the *ZKSCAN1*-flanked constructs but this was not statistically significant (Figure 1F). RNase protection detected the 12→10 backsplice junction in both total and RNase R-treated human hippocampal RNA, demonstrating the presence of endogenous human brain 12→10 tau circRNA.

The tau circRNAs were originally cloned from human brain tissue using a PCR approach, and the characterization of the 12→10 circRNA using RT–PCR confirmed the predicted sequence (8). To further characterize the 12→7 circRNA, we used RT–PCR with primer sets spanning the backsplice exon junction. We detected the predicted PCR products in RNAs transfected with either the *ZKSCAN1* or tau expression constructs, ruling out alternative splicing events in the 12→7 circRNA (Figure 1G). The sequence of the tau circRNA was further confirmed by RNAseq experiments (Figure 6).

The data confirm that *MAPT* backsplicing from exons 12→10 and 12→7 occurs physiologically in human brain. The formation of the tau circRNAs can be modeled using expression constructs containing a minimum sequence consisting of exons 7–12 or exons 10–12 spliced together flanked by 1 kb of *MAPT* intronic sequence, which indicates that this region is sufficient to form tau circRNAs.

### The 12→7 tau circRNA is translated using its single start codon

**Figure 2.**
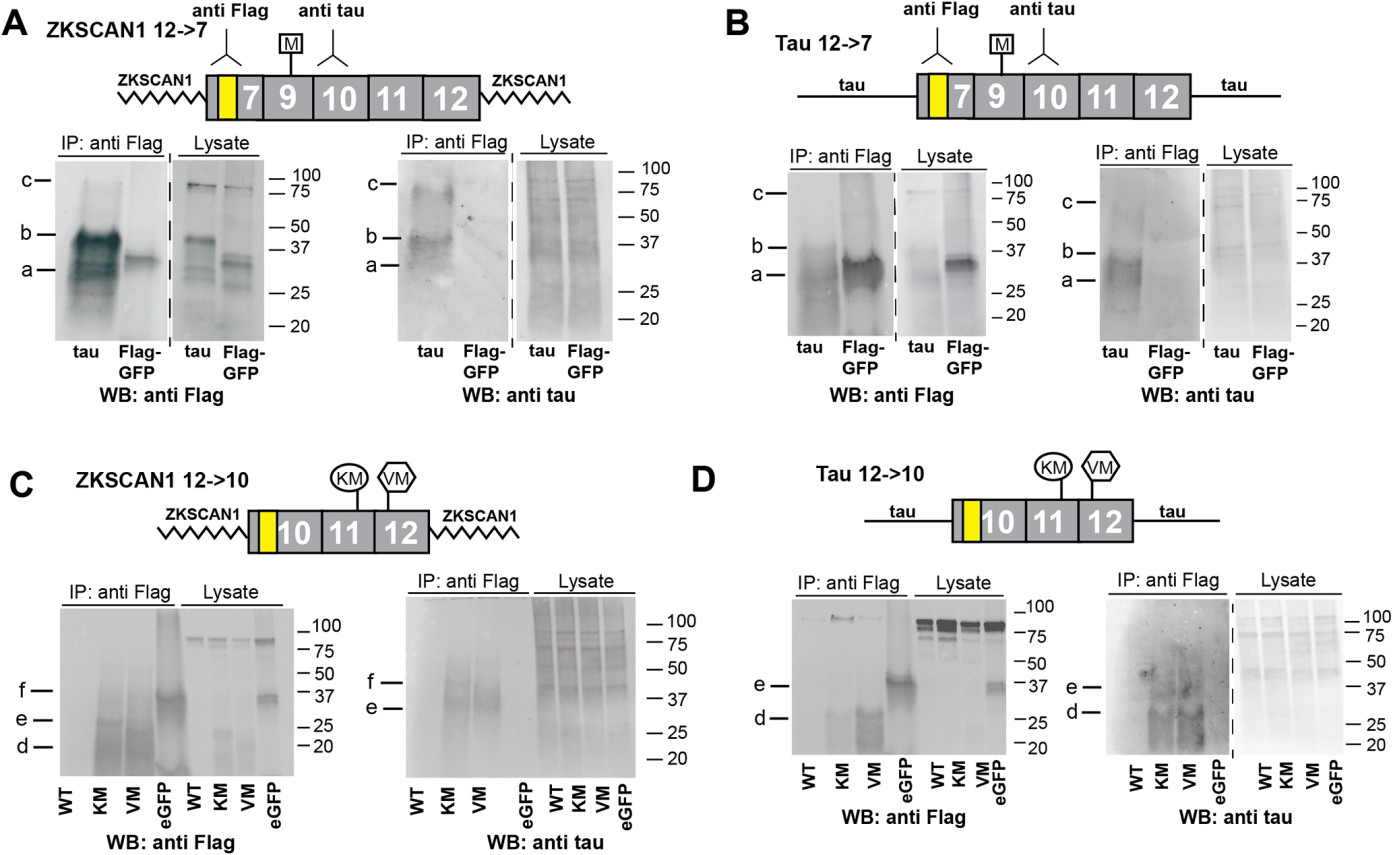
Tau circRNAs containing a start codon are translated. HEK293T cells were transfected with the expression constructs indicated. Cells were lysed 4 days post-transfection, and boiled lysates were immunoprecipitated using anti-Flag antibodies, as outlined in Supplementary Figure S2. The location of the antibody epitopes is indicated. (**A**) Transfection of 12→7 constructs flanked by *ZKSCAN1* introns. (**B**) Transfection of 12→7 constructs flanked by the wild-type *MAPT* introns. (**C**) Transfection of 12→10 constructs flanked by the *ZKSCAN1* introns. The wild-type construct does not contain start codons; the locations of the FTLD-tau mutations introducing start codons are schematically indicated. (**D**) Experiment similar to (C), with the cDNA flanked by wild-type *MAPT* introns.

The 12→7 tau circRNA contains one start codon in exon 9 that is in the open reading frame (ORF) and could act as a potential start codon for the 12→7 circRNA. We thus asked whether this circRNA can be translated by transfecting 12→7 tau circRNA expression constructs into HEK293T cells. Each construct contained a 3× Flag tag that precedes the start codon, so that only the tau circRNA can express Flag-tagged protein. Protein was immunoprecipitated after 4 days of transfection, using boiled cell lysates as circ tau proteins are heat stable, similar to the linear tau proteins (39) (Supplementary Figure S2). The circ tau protein was detected by using an anti-Flag as well as an anti-tau antiserum against exon 10 sequences in western blots. Green fluorescent protein (GFP) with a 3× Flag tag was used as an immunoprecipitation control (Figure 2A, B).

We detected protein expression that was stronger when the tau cDNA was flanked by *ZKSCAN1* introns compared with the wild-type *MAPT* introns, which reflects the RNA expression (Figures 1D, F and 2A, B). Independent of the flanking introns, we detected two strong bands of ∼30 and ∼40 kDa and a fainter protein of ∼80 kDa, termed proteins a–c (Figure 2A, B). The multiple bands are indicative of a rolling circle translational mechanism seen in other circRNAs (15). The expressed protein sizes correspond to translation starting at the single methionine in exon 9, which pauses for the first time at the site of initiation after one round, generating peptide ‘a’ of 27 kDa. The rolling circle translation continues, adding ∼14 kDa of protein, generating peptide ‘b’, which indicates translational stalling in exon 12. Another round of translation generates peptide ‘c’ of ∼80 kDa (Supplementary Figure S1D). All tau circRNA expression clones generate a ‘smear’ between the major bands, which probably indicates numerous translational stops that could be caused by circRNA structures as well as differently phosphorylated proteins. Thus, the 12→7 tau circRNA can generate proteins which contain all four tau microtubule-binding domains, resembling fragments of the linear tau protein.

### The 12→10 circRNA is translated when two FTLD-TAU mutations introduce start codons

The 12→10 circRNA lacks a start codon, but also lacks stop codons and thus constitutes a 288 nt long ‘ORF’. As expected, when flanked by either the *ZKSCAN1* or wild-type *MAPT* introns, we do not observe a protein product (Figure 2C, D). Two mutations in *MAPT*, K317M (36) and V337M (37,38), located in exons 11 and 12, cause FTLD-Tau via unknown molecular mechanisms. These mutations would introduce start codons to the 12→10 circRNA ORF. We introduced these mutations into our start codon-less 12→10 tau constructs and tested their influence on protein expression. As shown in Figure 2C and D, both K317M and V337M mutant constructs generated proteins and we observed three bands, d–f, that are detected by antisera against either the Flag-tag or tau exon 10 sequences. Similar to the 12→7 tau circRNA, the multiple bands are indicative of a rolling circle translational mechanism. After one round, defined as translation from start codon to start codon, the predicted protein has a size of 12,945 Da. Bands e and f with a predicted size of 25 and 35 kDa immune-react with anti-Flag and anti-tau antisera and probably correspond to two and three rounds of rolling circle translation that stops at the translational initiation site (Supplementary Figure S1C). Similar to the 12→7 tau circRNA constructs, we observed a ‘smear’ in the protein bands, which could indicate multiple translational stops due to circRNA structure, as well as protein phosphorylation. The circ tau proteins were generated both in the *ZKSCAN1* and wild-type *MAPT* intron background, suggesting that translation occurs independently of the genomic context after tau circRNA formation.

The nature of the protein was further validated using mass spectrometry (MS). We analyzed trypsinated immunoprecipiates from 12→10 V337M expression constructs using LC/MS. The analysis confirmed the sequence of the predicted protein. Importantly, we identified peptides that contained the only start codon in the middle of its sequence (peptide #4, Supplementary Figure S3A–C), confirming that the protein was generated in a rolling circle formation. Since trypsin cuts exactly at the exon–exon junction, we employed a different protease, Asp-N, that cuts N terminal of aspartic or cysteine residues. Using this enzyme, we could detect the backsplice junction peptide (Supplementary Figure S3D). Thus, the 12→10 tau circRNA, which is most abundant in human brain, can be translated once a start codon is introduced through FTLD-Tau mutations, which suggests that these mutations might cause pathology through tau circRNAs.

### Adenosine to inosine RNA editing promotes translation of 12→10 tau circRNA lacking a start codon

The molecular mechanism leading to circRNA translation (15) and specifically tau circRNA translation is enigmatic, as the circRNAs lack known ribosomal entry sites. Similar to most circRNAs, tau circRNAs are predominantly cytosolic (8). CircRNAs are more stable than their linear counterparts and form a rod-like structure (40), suggesting that they can form secondary structures that are the substrate of ADAR enzymes. There are three ADAR genes in humans, *ADAR1–ADAR3* (Hugo names *ADAR, ADARB1* and *ADARB2*), that deaminate adenosines to inosines after binding to double-stranded RNA. ADAR1 and ADAR2 proteins are catalytically active, whereas ADAR3 protein is inactive (41). To test the impact of ADAR activity, we first co-transfected the *MAPT* 12→10 wild-type constructs flanked by the wild-type *MAPT* introns with expression clones of ADAR1–ADAR3 and GFP.

**Figure 3.**
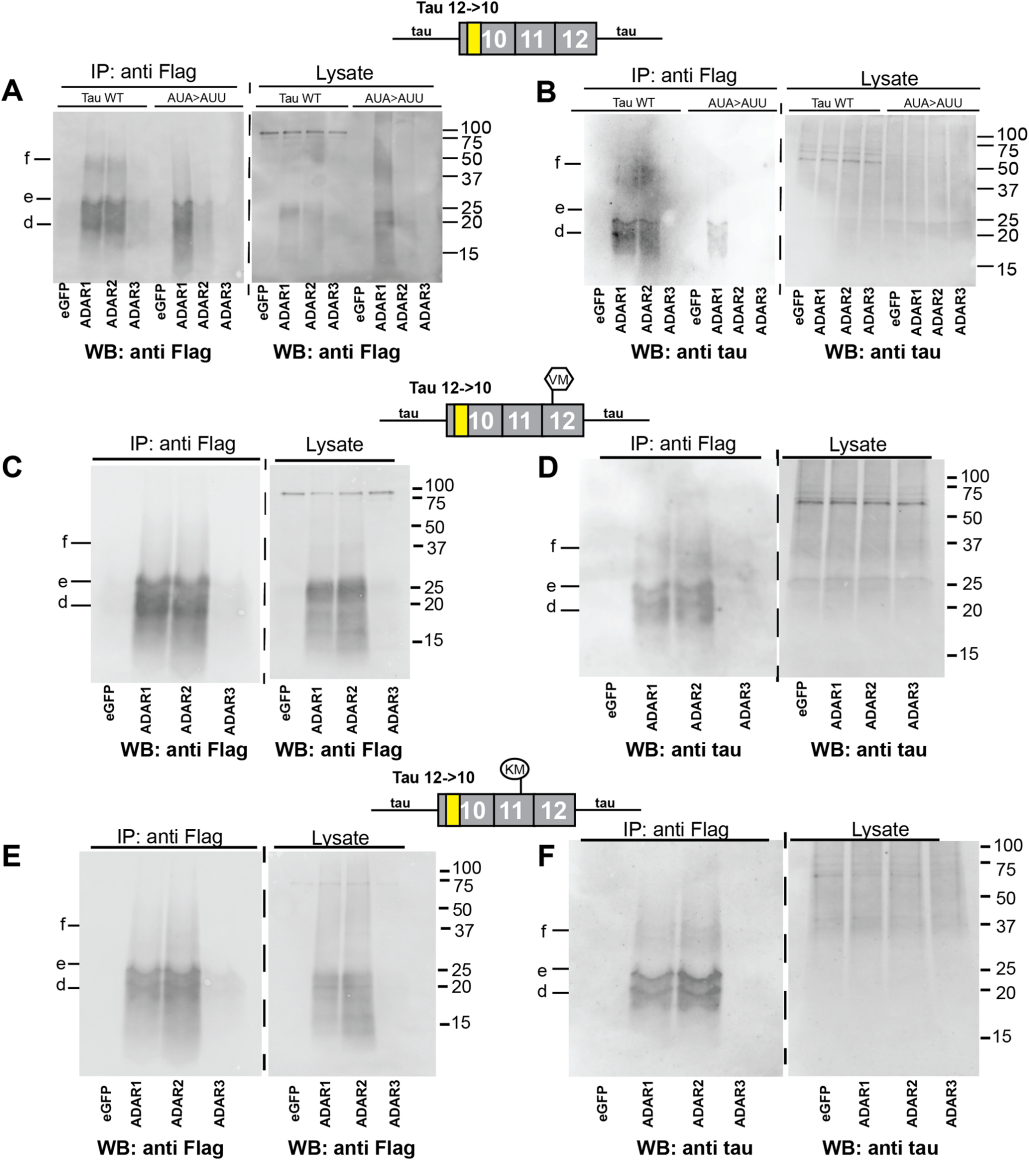
ADAR activity promotes protein expression from the 12→10 tau circRNA. Constructs expressing the 12→10 circRNA were co-transfected with ADAR enzymes in HEK293T cells. After 4 days of transfection, protein was isolated through anti-Flag immunoprecipitation and detected by western blot using anti-Flag and anti-tau antisera. (**A**) The 12→10 wild-type and AUA>AUU constructs were co-transfected with GFP, ADAR1, ADAR2 and ADAR3 expression constructs. In AUA>AUU, two AUA codons are changed to AUU (Ile→Ile) to prevent RNA editing. The protein was immunoprecipitated with anti-Flag and detected with a Flag antiserum. (**B**) The protein from (A) was detected with anti-tau antiserum. (**C**) The 12→10 V337M mutant was co-transfected with GFP and ADAR1–ADAR3. Protein was isolated through anti-Flag immunoprecipitation and detected with anti-Flag antisera. The signal can also be detected in the eGFP-transfected cells, as shown in Figure 2D. (**D**) The protein from (C) was detected with anti-tau antiserum. (**E**) The 12→10 K317M mutant was co-transfected with GFP and ADAR1–ADAR3. Protein was isolated through anti-Flag immunoprecipitation and detected with anti-Flag antisera. The signal can also be detected in the eGFP-transfected cells, as shown in Figure 2D. (**F**) The protein from (E) was detected with anti-tau antiserum.

In the presence of ADAR1 and ADAR2, a strong increase in circ tau protein expression was detected both with anti-Flag and with anti-tau antisera, despite the lack of a canonical start codon (Figure 3A, B). The increase in protein expression allowed detection in crude lysates using the Flag antibody (Figure 3A). We were not able to detect protein expression in lysates without co-transfected ADAR activity (Figure 3A, GFP). The 12→10 *MAPT* circRNA ORF has two in-frame AUA codons and we hypothesized that AUI could be a start codon, as inosines preferentially base-pair with cytosines, i.e. are read as guanosine, making the AUI codon a potential start codon, similar to AUG (42). We changed both AUA codons to AUU (Ile–Ile) and tested the influence of ADARs on protein expression from these variants. We found a strong reduction in ADAR2’s ability to promote translation, but saw no change when ADAR1 was used. This indicates that ADAR2 activates translation of 12→10 tau circRNA by changing AUA to AUI, which could act as start codons (Figure 3A, B, AUA>AUU).

The overexpression of ADAR1–ADAR3 was confirmed by analyzing RNAseq data from transfected cells and by western blot of transfected cell lysates (Supplementary Figure S4A, B). The data confirmed the predicted increase in ADAR1–ADAR3 expression, which is stronger for ADAR2 than ADAR1 and weakest for ADAR3. RNAseq showed that ADAR3 is expressed at very low levels in HEK293T cells. Importantly, ADAR enzyme activity is regulated by phosphorylation (43), suggesting that the amount of protein detected by western blot or the amount of mRNA determined by RNAseq does not necessarily correlate with an increase in activity.

### Adenosine to inosine RNA editing promotes the translation of tau circRNAs carrying the V337M and K317M mutations

Due to the relationship to human disease, we next tested the effect of adenosine to inosine editing on the FTLD-Tau mutations V337M and K317M in the 12→10 tau circRNAs flanked by *MAPT* introns (Figure 3C–F). Again, with these constructs, we saw a dramatic increase in protein production when ADAR1 and ADAR2 were co-transfected, whereas the negative controls GFP and ADAR3 had no effect.

For comparison, we tested the effect of ADAR1–ADAR3 on linear mRNA substrates. We generated a 3× Flag-tagged 0N4R linear *MAPT* expression construct that we co-transfected with ADAR1–ADAR3 and the GFP control. The protein expression was analyzed using boiled cell lysates containing the same amount of protein, employing an anti-Flag antiserum. As a loading control, we used a calreticulin antibody, because, similar to tau protein, calreticulin is heat stable (Supplementary Figure S4C, D). For the FLAG-0N4R, there is a slight increase of protein expression with ADAR1 and ADAR3 that is much weaker than the increase seen with the circRNAs. This ADAR effect cannot be detected for calreticulin.

These data suggest that A>I editing promotes translation of circular RNAs and also can generate non-canonical start codons. Thus A>I editing could be a new translational mechanism acting on circRNAs.

### Adenosine to inosine RNA editing promotes translation of 12→7 tau circRNA

**Figure 4.**
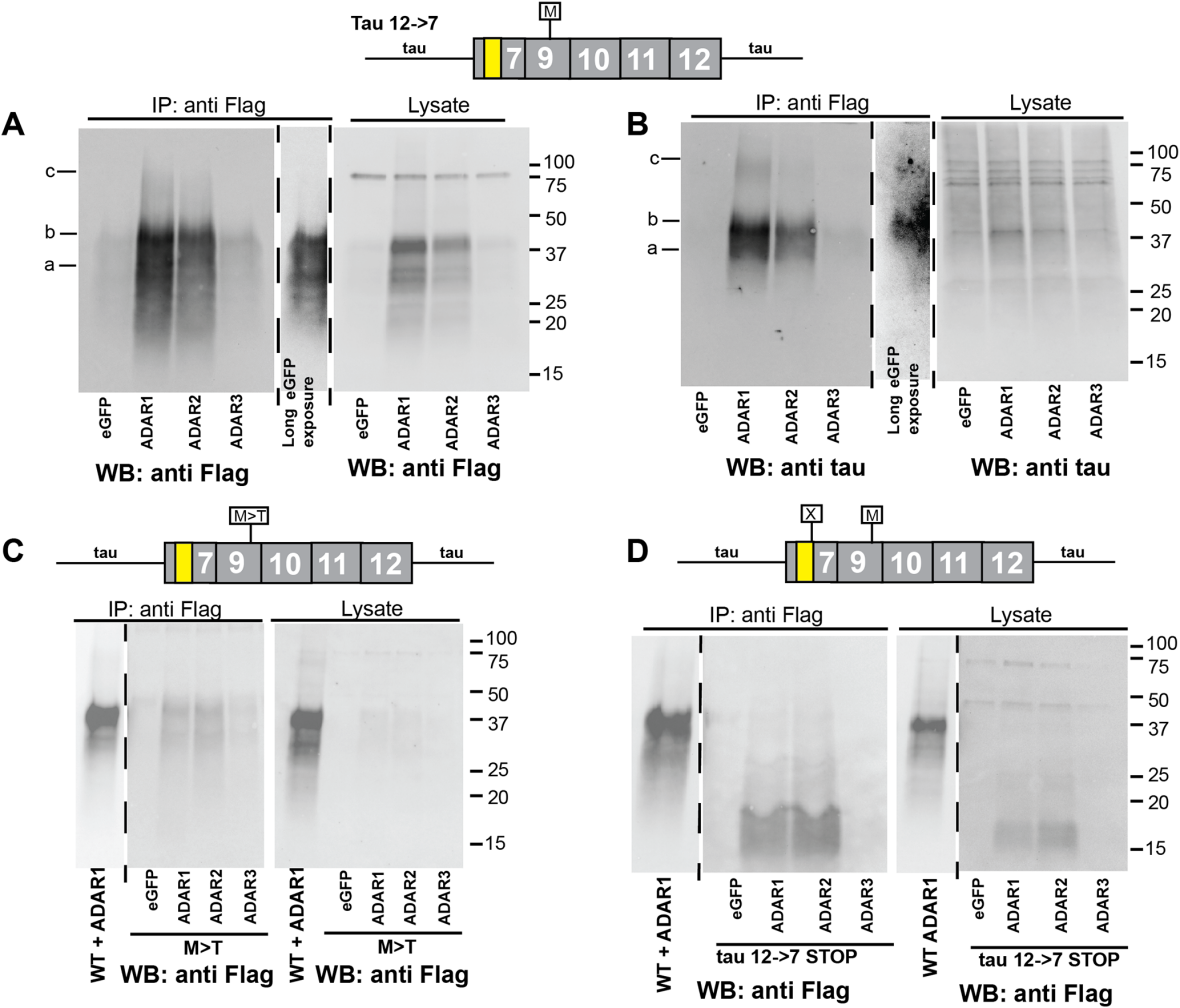
ADAR activity promotes translation of tau circRNAs. HEK293T cells were co-transfected with the tau 12→7 circRNA expression constructs indicated on top and the ADAR variants shown at the bottom of each blot. (**A**) Wild-type 12→7 circRNA expression clones were co-transfected with GFP, ADAR1, ADAR2 and ADAR3 expression constructs. Protein was isolated after 4 days using anti-Flag immunoprecipitation and detected by western blot using anti-Flag. (**B**) A similar experiment to that in (A), but the detection was with anti-tau. (**C**) The 12→7 construct where the single start codon was removed through mutation (M>T) was co-transfected with GFP, ADAR1, ADAR2 and ADAR3 expression constructs, immunoprecipitated and detected with anti-Flag. The exposure time of the wild-type 12→7 construct was one-fifth that of the mutant constructs. (**D**) The 12→7 construct with a stop codon was co-transfected with GFP, ADAR1, ADAR2 and ADAR3 expression constructs, immunoprecipitated and detected with anti-Flag.

We next asked whether RNA editing also affects the 12→7 tau circRNA that naturally contains a start codon. As shown in Figure 4A and B, both ADAR1 and ADAR2, but not ADAR3, strongly promote protein translation from the 12→7 tau circRNA. Again, when ADAR activity is increased, we can detect circ tau protein in the lysates using Flag antibodies.

To determine the effect of A>I editing on another substrate lacking a start codon, we tested ADAR activity on the start codon-less mutant AUG>ACG (M>T) of the 12→7 tau circRNA. We co-transfected the 12→7 M>T expression construct with GFP and ADAR1–ADAR3, and found translational activation of this circRNA through ADAR1 and ADAR2 activity. However, the level of translation does not reach that observed with the wild-type construct that contains the authentic start codon (Figure 4A, B).

To further validate the use of the single ORF in the 12→7 tau circRNA, we introduced a stop codon immediately downstream of the 3× Flag tag. Transfecting the corresponding expression constructs into HEK293T cells showed expression of two major protein bands at 15 and 20 kDa, which correspond to the predicted peptide of 15.5 kDa and a probably phosphorylated form (Figure 4D).

For comparison with an unrelated circRNA, we tested Upf1 circRNA containing a start codon in co-transfection with ADAR1–ADAR3 and again saw a large increase in translation when ADAR1 or ADAR2 was present (Supplementary Figure S5).

These data indicated that RNA editing strongly increases translation of tau circRNAs, probably by promoting circRNA interaction with the ribosome and possibly by acting in support of ribosomal entry. A>I editing also changes encoded amino acids, allowing translation of the start codon-less 12→10 circRNA.

### Proteins made from the 12→7 circRNA constructs are the result of backsplicing

**Figure 5.**
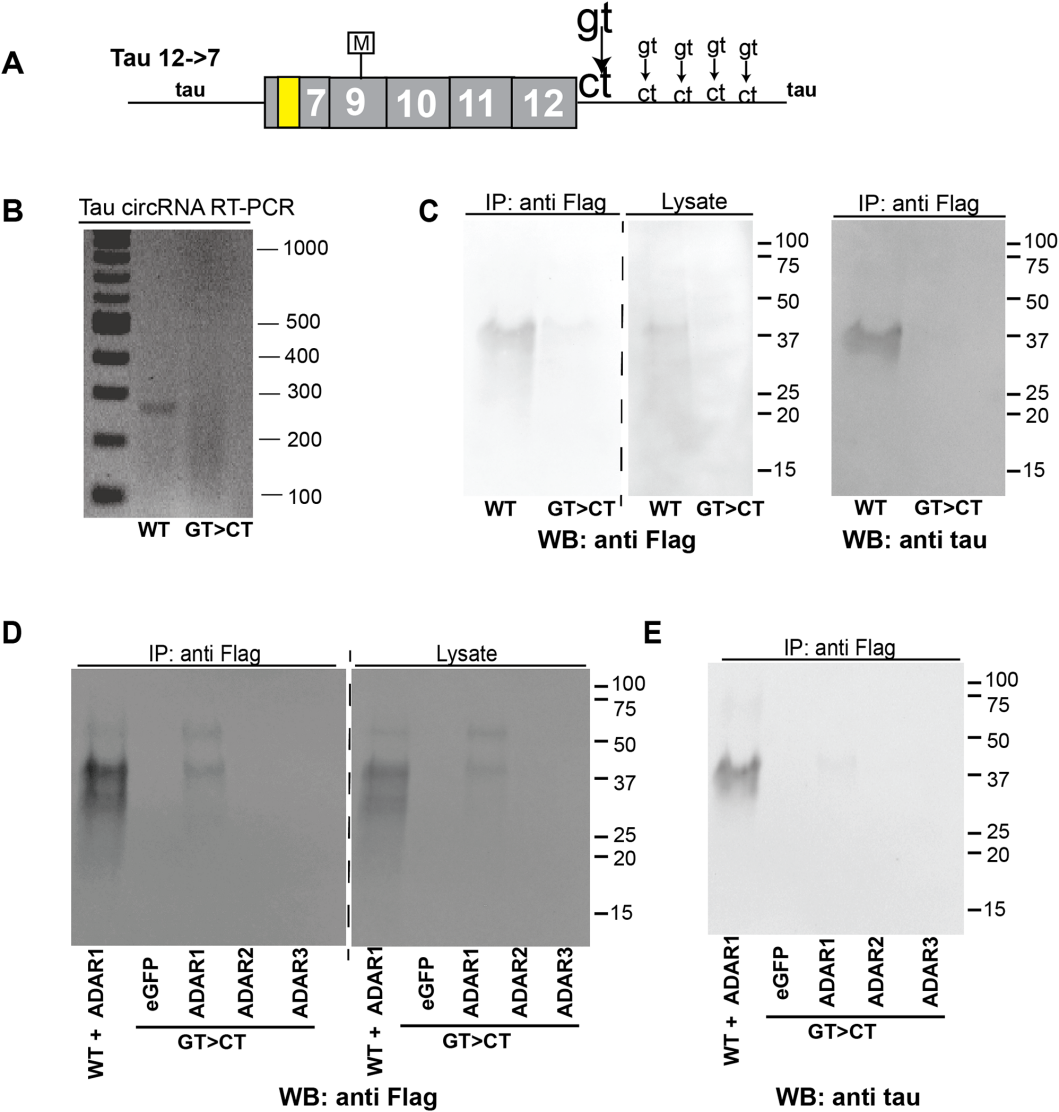
Tau circRNA formation depends on backsplicing. (**A**) The gt of the 5′-splice site (large letters) and four cryptic gt dinucleotides downstream of the authentic splice site (small letters) were mutated to ct. (**B**) The 12→7 wt and gt>ct mutants were transfected into HEK293T cells, and circRNA expression was detected using RT–PCR, employing the f2/r2 primer set (Figure 1G). (**C**) The 12→7 wild type and gt>ct mutant were co-transfected with GFP in HEK293T cells and protein was detected by western blot after immunoprecipitation. (**D**) The 12→7 ct mutant was co-transfected with ADAR1–ADAR3 expression clones into HEK293T cells. Protein was isolated using Flag immunoprecipitation and detected using anti-FLAG antisera. (**E**) A similar experiment to that in (D), but detection was with anti-tau.

**Figure 6.**
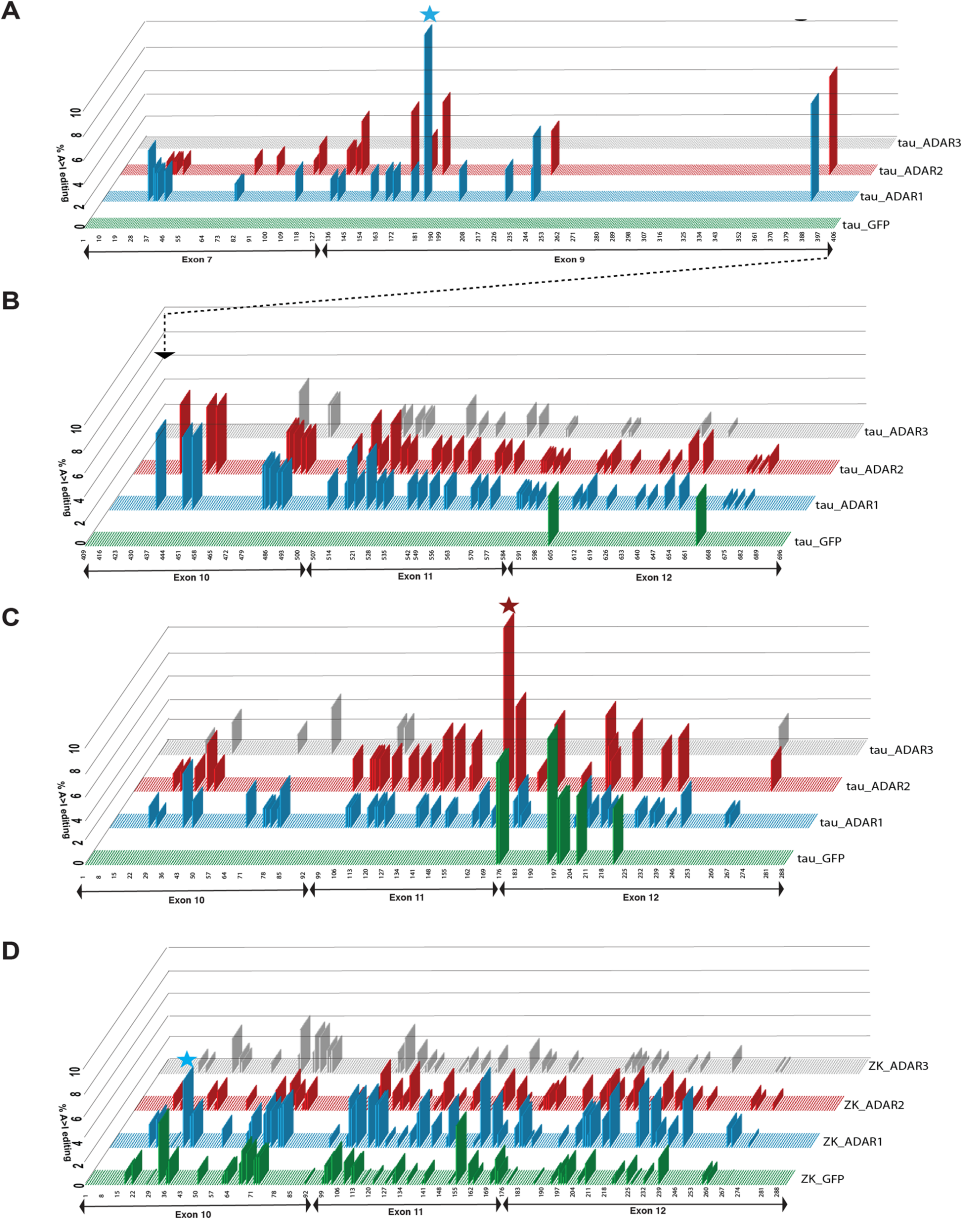
RNA editing sites of tau circRNAs depend on the flanking introns and neighboring sequences. The 12→7 and 12→10 wild-type tau expression constructs were co-transfected into HEK293T cells with expression clones of GFP, ADAR1, ADAR2 or ADAR3 as indicated. CircRNA was sequenced, and the editing sites were calculated as A>G changes. The 12→7 tau circRNA is represented in two panels to allow alignment of its exon 10 to exon 12 part with the 12→10 tau circRNA (direction is indicated by an arrow). Stars indicate the site of highest editing. (**A**) 12→7 exons 7 and 9, (**B**) 12→7 exons 10–12, (**C**) 12→10 flanked by endogenous tau introns, (**D**) 12→10 flanked by *ZKSCAN1* introns.

To further confirm that the tau proteins were generated from circRNAs, we mutated the first nucleotide of the intron in the 5′ backsplice site, gt→ct. A single mutation still results in protein production, as there are three gt dinucleotides 9, 18 and 36 nt downstream of the authentic splice site (Figure 5A). Usage of these splice sites will generate a circRNA with an ORF. In addition, there is a fourth gt that would cause a frameshift if used. We were not able to detect exonic sequences corresponding to the use of these three splice sites after transfection of the wild-type construct using RNAseq (Figure 6), suggesting that these cryptic splice sites are only activated once the natural site is mutated. To analyze the backsplicing, we mutated these four sites gt>ct. First, we used RT–PCR to determine circRNA expression from this gt>ct construct. We could detect circRNA expression from the wild type containing the authentic gt splice site, but not the mutant with the authentic and cryptic splice sites mutated (Figure 5B). Next, we determined protein expression from these clones by co-transfecting them with GFP and could detect only weak background signal when compared with the wild type (Figure 5C). To determine an effect of ADAR activity on the gt>ct mutants, we co-transfected them with expression clones for GFP and ADAR1–ADAR3. We observed a drastic reduction in protein that could not be detected in the GFP-, ADAR2- and ADAR3-expressing cells. There was a weak signal in ADAR1 co-transfection, possibly caused by cryptic splice sites in exon 12 (Figure 5D, E).

In summary, the formation and subsequent translation of the 12→7 tau circRNAs depends on backsplicing using the 5′-splice site of exon 12.

### RNA editing of tau circRNAs depends on neighboring sequences and flanking introns

We next asked which sites are edited in the tau circRNAs. We co-transfected tau circRNA expression constructs with expression clones for either GFP, ADAR1, ADAR2 or ADAR3 in HEK293T cells and analyzed the RNAs using RNAseq. Using the backsplice junction reads, we reconstructed the circRNAs and determined RNA editing by calculating the changes of adenosines to guanosines, as an inosine is read as a guanosine during cDNA synthesis. The quantification is tabulated in Supplementary Figure S6.

First, we analyzed the 12→7 construct flanked by the wild-type *MAPT* introns (Figure 6A, B). In this circRNA, a total of 141 residues are edited, 60 by ADAR1, 58 by ADAR2 and 21 by ADAR3. However, at each given site, the editing frequency was low, usually ∼3%. Editing is most prevalent in exons 10, 11 and 12. The sites that were least edited are found in exon 9. The highest degree of editing was found at residues 176 in exon 9, which is still much lower than editing of most linear mRNAs. For example, the glutamate receptor-B (GRIA2) mRNA is >90% edited at the specific editing site (44). There are two clusters of editing sites in exon 10 (residues 472–480) and exon 12 (residues 662–671) which could lead to translational stops, as a run of inosine causes ribosome stalling (42).

Next, we analyzed the 12→10 expression constructs flanked by its wild-type tau introns (Figure 6C). About half (49) of the 97 adenosines can be converted into inosines, 44 by ADAR1, 33 by ADAR2 and 7 by ADAR3. Similar to the 12→7 circRNA, the editing of individual sites was incomplete and, in most sites, <3% of adenosines were edited. Only one site (#219) was edited in 16% of the reads. As predicted by our mutational analysis, ADAR2 edits A#168 to inosine, which generates an AUI start codon that we confirmed using mutagenesis. In addition, there are two clusters (#97–99 and #195–198) of RNA editing sites located in exons 11 and 12, that could lead to ribosome stalling and hence stop the rolling circle translation (42).

Finally, we determined A>I editing in the 12→10 construct flanked by *ZKSCAN1* introns. Overall, we see 165 editing sites, 59 affected by ADAR1, 58 by ADAR2 and 46 by ADAR3. Editing is strongly increased by ADAR1. A total of 59 sites are edited in the GFP controls due to endogenous ADAR activity (Figure 6D). Thus, when flanked by *ZKSCAN1* introns, more sites were edited to a lower degree than in the tau intron-flanked constructs. This indicated that at least part of tau circRNA editing occurs during pre-mRNA processing in the nucleus and is sensitive to intronic context. As ADAR proteins can be detected in both the cytosol and the nucleus, it is possible that there is additional cytosolic circRNA editing, which remains to be determined.

Using the RNAseq data, we determined the tau circRNA expression levels normalized for the constitutively expressed circHIPK3 (30). We found that co-transfection of ADAR1, ADAR2 and ADAR3 increased tau circRNA expression up to 6-fold when the tau expression clones were flanked by wild-type *MAPT* or *ZKSCAN1* introns (Supplementary Figure S7), suggesting that circRNA editing affects their expression levels.

In all systems, we observed A>I editing when only GFP expression clones were transfected, especially in the presence of the *ZKSCAN1* Alu elements, which is probably due to the endogenous ADAR1 and ADAR2 activity in HEK293T cells. Surprisingly, the A>I editing profile changed when we transfected the catalytically inactive variant ADAR3, which could indicate a change of RNA structure due to binding of ADAR3.

In summary, the exact A>I editing of circRNAs depends on the flanking introns, indicating that editing occurs during the circularization of the pre-mRNA. The differences between editing sites in the 12→10 and 12→7 constructs show that long-range interactions, for example between exon 7 and exon 10, contribute to the double-stranded RNA structures necessary for A>I editing. As an inosine is interpreted as a guanosine by the ribosome (42), ADAR activity not only promotes translation, but also changes the amino acid sequence encoded by the circRNAs. Thus, it is possible that tau circRNAs encode a mixture of proteins.

### Tau circRNAs from group I introns are translated

**Figure 7.**
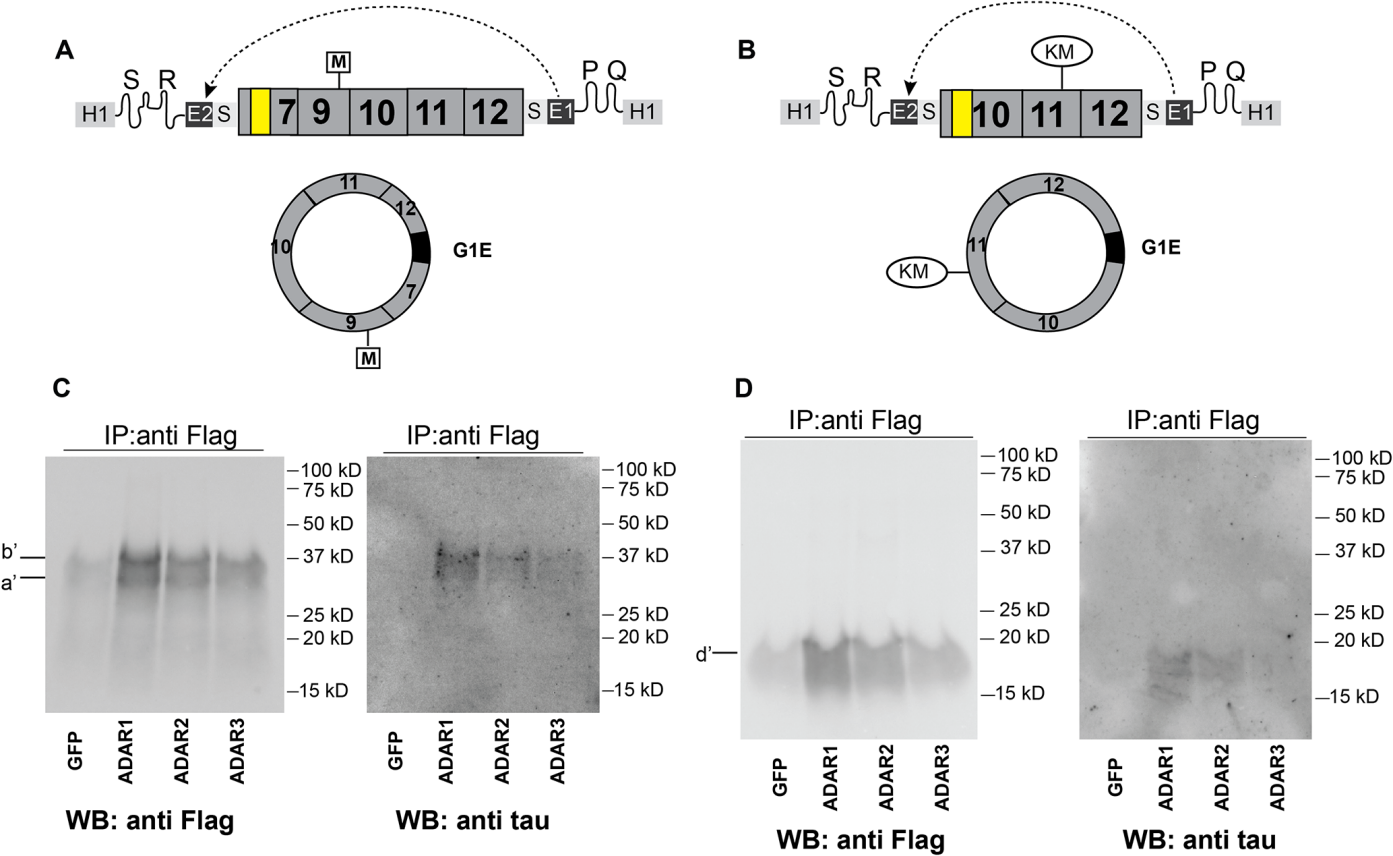
Group I introns form tau circRNAs that are translated. (**A**) Structure of the group I intron constructs expressing tau circRNA 12→7 *in vitro*. The Anabaena pre-tRNA self-splicing group I intron order was reversed, Exon 2 (E2) is now preceding Exon 1 (E1). The group I catalytic core consisting of SR, PQ helices is indicated. The tau circRNA is cloned as an ‘intron’ between these exons and will be spliced out as a circle, similar to all group I introns. G1E: group 1 exons (24 nt). (**B**) Predicted structure and self-splicing of the tau circRNA 12→10 K317M. (**C**) The 12→7 group I intron expression construct was co-transfected into HEK293T cells with either GFP, ADAR1, ADAR2 or ADAR3 expression clones. Protein was isolated using anti-Flag immunoprecipitation and detected by western blot using anti-Flag and anti-tau antisera. (**D**) The K317M 12→10 group I intron expression construct was co-transfected with the ADAR constructs indicated or GFP as control into HEK293T cells. Protein was isolated using anti-Flag immunoprecipitation and detected by western blot using anti-Flag and anti-tau antisera.

Hypothetically, pre-mRNAs could form concatemers due to *trans*-splicing that will show backsplicing junction sequences. To rule out translational effects from such theoretically *trans*-spliced RNAs, we expressed tau 12→7 and 12→10 circRNA from a self-splicing group I intron (45). We used a previously described expression system where the RNA to be circularized is cloned as an intron between inverted group I exons (46–48) (Figure 7A, B). The group I intron-derived circRNAs contain short additional sequences from their flanking exons (G1E, Figure 7A, B). Expression of the 12→7 and 12→10 circRNAs from group I introns in HEK293T cells generates circRNAs that are translated showing the same dependency on ADAR activity as the tau constructs undergoing backsplicing (Figure 7C, D).

We thus observed ADAR-dependent circRNA translation in two independent systems: regular pre-mRNA circular splicing and group I self-splicing, which together rule out that *trans*-splicing generates a linear RNA that mimics a circRNA.

### Proteins translated from tau circRNA form aggregates similar to neurofibrillary tangles (NFTs)

Linear tau protein aggregates into NFTs when incubated with poly anions *in vitro* for several days; for example, heparin is used to promote formation of aggregates that can be visualized using electron microscopy (49). Importantly, NFTs also form in the presence of RNA (34). Circ tau proteins contain multiple microtubule-binding motifs and are thus structurally similar to the K18 tau peptide that is sufficient to promote NFT formation (34).

We thus tested the ability of proteins encoded by tau circRNAs to aggregate *in vitro*. We expressed circ tau protein in HEK293T cells and, after immunoprecipitation, eluted the protein with a 3× Flag peptide. In order to increase the amount of circ tau protein, ADAR1 was co-transfected. Since the kinase DYRK1A increased tau self-aggregation *in vitro* (50), we also tested circ tau protein made in the presence of DYRK1A.

**Figure 8.**
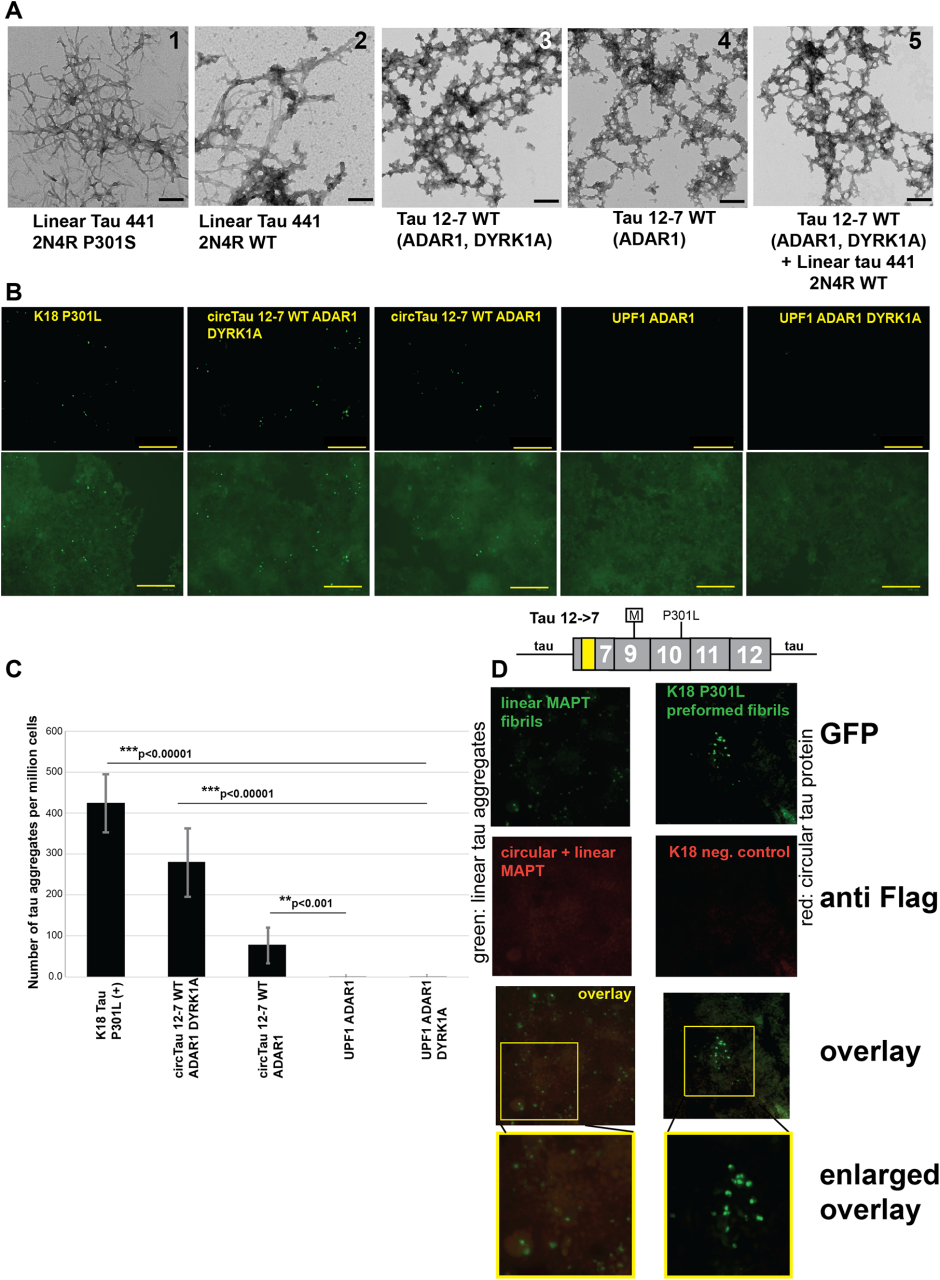
Tau circRNAs promote tau fibril formation. (**A**) Linear tau and tau circular proteins were assembled *in vitro* and visualized by TEM. Aggregation was initiated through heparin for linear tau isoform 441 2N4R P301S and by yeast tRNA with all other samples. The scale bar is 200 nm. (**B**) Proteins indicated on top were introduced in tau biosensor cells expressing YFP-tau repeat domain P301S and tau repeat domain P301S-CFP. Aggregation of the YFP and CFP tau repeat domain P301S proteins is detected as a green fluorescence. K18 P301L is the K18 peptide encompassing the microtubule-binding domains (34) carrying the P301L FTLD mutation. The scale bar is 200 μm. (**C**) Quantification of the tau aggregates formed in (B). (**D**) Co-staining of tau aggregates and circular tau proteins. Aggregates in tau biosensensor cells were generated by incubating them with pre-formed fibrils made from 12→7 P301L tau protein and linear P301S tau monomers. The green GFP signal indicates the aggregation of MAPT and the red signal is anti-FLAG, used to detect the protein from the circRNA. The enlarged area shows FLAG immunoreactivity in the tau aggregates (yellow staining). Commercial K18 P301L fibers without circRNA were used as a positive control for aggregate formation and a negative control for Flag staining.

The aggregates were first analyzed using transmission electron microscopy (TEM). As a positive control, we used commercially available linear 2N4R tau fibrils carrying the FTLD-tau mutation P301S that were aggregated in the presence of heparin (Figure 8A-1). We next formed aggregates of linear 2N4R tau (WT) by incubating 50 ng/μl recombinant protein with 25 ng/ml yeast tRNA for 4 days which gave similar fibrils (Figure 8A-2). We then incubated 12→7 circ tau protein generated after co-transfecting 12→7 expression clones with DYRK1A and ADAR1 with 500 ng/ml yeast tRNA and observed fibrils that were thicker and shorter than fibrils seen with linear tau (Figure 8A-3). We repeated the experiment with circ tau protein isolated from cells co-transfected with the tau circ 12→7 expression construct without the kinase DYRK1A and observed similar aggregates (Figure 8A-4). Finally, circ tau protein was incubated with 2N4R tau linear protein at a 1:1 molar ratio in the presence of 500 ng/ml yeast tRNA for 4 days, which again resulted in shorter and thicker fibrils (Figure 8A-5).

The aggregation of tau protein can be observed in biosensor cells that express linear tau protein fused to cyan fluorescent enhanced protein (eCFP) and yellow enhanced fluorescent protein (eYFP), which results in a green signal upon aggregation (51). Pre-formed fibrils, made from either the commercially available K18 peptide or from 12→7 expression clones co-transfected with ADAR1 and DYRK1A or only ADAR1, were added to tau biosensor cells using Lipofectamine (51). Tau aggregates emerged after 2 days and were quantified after 4 days in culture. In each of these cases, we observed the formation of tau proteinaceous aggregates indicated by green dots (Figure 8B). We used circular UPF1 (Supplementary Figure S5) isolated from cells transfected with ADAR1 and ADAR1/DYRK1A as a negative control. Quantification of the number of dots showed a statistically significant increase of aggregates in the cells treated with circ tau protein (Figure 8C), especially when DYRK1A is present.

We next used this system to determine the localization of proteins from tau circRNAs in biosensor cells. We incubated the cells with *in vitro* pre-formed fibrils made from 12→7 P301L circular tau protein and linear P301S tau monomers. The use of the P301L mutant increased the number of tau aggregates. The tau circular protein was detected using its Flag tag. We see immunoreactivity throughout the cells and, in some but not all tau aggregates, the tagged circular tau protein can be detected within the aggregate (Figure 8D).

Thus, circ tau proteins are associated with tau aggregation in human reporter cells, comparable with a synthetic peptide (K18) that corresponds to the microtubule repeat regions. In summary, we found that the human *MAPT* gene generates novel proteins from its circRNAs that potentially contribute to tau aggregation and NFT formation. The translation of these tau circRNAs is regulated by epigenetic adenosine to inosine RNA editing.

## DISCUSSION

### Use of a novel reporter system to analyze circRNA function

CircRNAs have been identified in numerous eukaryotes, showing widespread expression at generally very low levels. Functionally, circRNAs have been implicated as microRNA sponges (52) and transcriptional regulators (53). Since most circRNAs contain ORFs, they could be templates for proteins (54). However, their low expression levels and lack of a general clear function also suggest that circRNAs could be mere byproducts of pre-mRNA processing (55).

We therefore studied two tau circRNAs that we previously identified in human brain (8). Employing RNase protection analysis, we validated their physiological expression and, using reporter systems, we found that tau circRNAs are translated, and that circRNA translation is strongly promoted by A>I RNA editing. In these novel circRNA reporter genes, we removed large *MAPT* introns to facilitate the analysis. The resulting circRNAs will thus contain only one exon junction complex at the backsplice junction, but will lack other exon junction complexes, which could interfere with translational regulation and circRNA stability. Furthermore, to show the circRNAs’ physiological functions and especially their *in vivo* RNA editing, our proof of principle studies need to be followed up by analyzing brain *MAPT* circRNAs which, due to their low abundance, will require isolation of the circRNAs prior to RNAseq.

### Tau circRNAs are translated

The translated circ tau proteins are predicted to encode multimers of microtubule-binding sites found in the linear tau. In the 12→7 circRNA, the circ tau proteins start from a single in-frame start codon in exon 9. The 12→10 tau circRNA does not contain a start codon, but A>I RNA editing introduces start codons. Using mutagenesis and RNAseq, we identified a single codon, AUA (#99) in exon 11 that is changed to AUI by ADAR2, which probably serves as a start codon. We did not identify a similar start codon for ADAR1, which opens up the possibility that ADAR1 generates different start codons, which as yet are unknown. Using RNAseq, we detected four consecutive editing sites in exon 12. Runs of 3–4 inosines are known to cause ribosome stalling in mRNAs (42), suggesting that translation is partially terminated at these sites, adding to the termination at the translational start site. However, the exact stop sites need to be determined.

As both tau circRNAs lack stop codons and translation can progress repeatedly around the circular structure, they express multiple protein bands, which have also been seen in other systems (15). In addition, we see a ‘smear’ between the bands. To further investigate the cause of this heterogeneity, we blocked ubiquitinylation using the inhibitor MG132 and found a strong increase of detectable protein in the presence of MG132, but still a ‘smear’ of bands, suggesting that the smearing could be due to translational stops caused by RNA secondary structures and that circ tau proteins are subject to proteasomal degradation (Supplementary Figure S8)

### Tau circRNAs undergo widespread but incomplete adenosine to inosine RNA (A>I) editing

Using RNAseq, we analyzed the circRNA editing profile after we co-transfected tau circRNA expression constructs with ADAR-expressing constructs. We observed widespread RNA editing that was, however, incomplete at each individual site, which is in contrast to well-documented A>I editing in mRNAs. Since A>I editing depends on double-stranded RNA structure, the incompleteness suggests that tau circRNAs rapidly oscillate between various RNA structures, which will result in differentially edited circRNAs. This heterogeneity could generate terminating RNA sequences or structures at various locations. Thus, ribosomal stalling could occur at various locations in the circRNA, possibly explaining the smear in circ tau protein due to different stops.

The most striking effect of ADAR activity is the strong increase in translated circ tau protein. Using RNAseq, we quantified the expression levels of tau circRNAs after co-transfecting their expression constructs with ADAR1–ADAR3. We observed an up to 6-fold increase in circRNA. The increase in circRNA expression is also observed for ADAR3 that has no effect on protein translation, suggesting that the influence on translation is independent of the increase in circRNA levels. A mislocalization of ADAR2 influenced translation levels through eIF2 signaling pathways in a model of amyotrophic lateral sclerosis (56), and we therefore tested the effect of ADAR1–ADAR3 activity on linear tau protein. Co-transfection of linear tau and ADAR1–ADAR3 expression constructs showed a slight effect of ADAR1 and ADAR3 on linear tau protein and no effect on the unrelated calreticulin protein expression levels. It is thus possible that ADAR activity influences translation of a subset of RNAs, with circRNAs showing the strongest ADAR-dependent increase in translation. The molecular mechanism for this increase remains to be determined. As circRNAs show widespread A>I editing, the existence of inosine-binding proteins that facilitate ribosomal entry could explain the increase in translation. However, such inosine-binding proteins remain to be identified.

### Circ tau proteins may contribute to primate-specific brain function

We have not detected corresponding tau circRNAs in rodents using RT–PCR (8), possibly because rodents lack Alu elements. Thus, the circ tau proteins are likely to be human or primate specific. We thus speculate that the 12→7 circ tau protein may have a physiological function in the primate brain. In several systems, brain complexity correlates with the number of RNA isoforms. For example, A>I editing in cephalopods occurs predominantly in their nervous system and probably contributes to their brain function (57). Alu elements in general promote circRNA formation (30), expanded during primate evolution and their number correlates with primate brain complexity (24). It is thus possible that A>I editing acting on circRNAs contributes to the advanced primate brains. However, in the *MAPT* system, other transposon sequences, Charlie 15a and MER5A/5B, flank the backsplicing sites and their contribution to pre-mRNA structures remains to be determined.

The comparison of A>I editing using constructs flanked by either natural tau or heterologous *ZKSCAN1* Alu elements showed widespread differences, suggesting that, similar to mRNA editing (44), the editing starts in the nucleus and is dependent on intronic regions that determine the pre-mRNA structure. The dependency on genomic context could contribute to variations seen between different *MAPT* haplotypes (58).

### FTLD-Tau mutations could act through circRNAs

For most FTLD-Tau mutations, the mechanism of action is incompletely understood (2), but they may act at least in part through the tau circRNA. Of note, known FTLD-Tau-inducing *MAPT* mutations do not introduce stop codons and each mutation keeps the reading frame. Moreover, 49/53 known mutation cluster in exons 7–13 and are thus either part of the tau circRNAs or could influence their generation through splice site competition. We tested two specific mutations, K317M and V337M, and found that they promote translation of the 12→10 tau circRNA. Mapping of the A>I RNA editing sites in the 12→10 tau circRNA showed widespread editing that changes at least 52/96 amino acids, but only two of these changes affect the same amino acids as the FTLD-Tau mutants N296H and K317M. We speculate that A>I editing leads to widespread low-degree amino acid changes in tau circular protein.

### Tau circRNA could contribute to Alzheimer's disease after activation of ADAR

**Figure 9.**
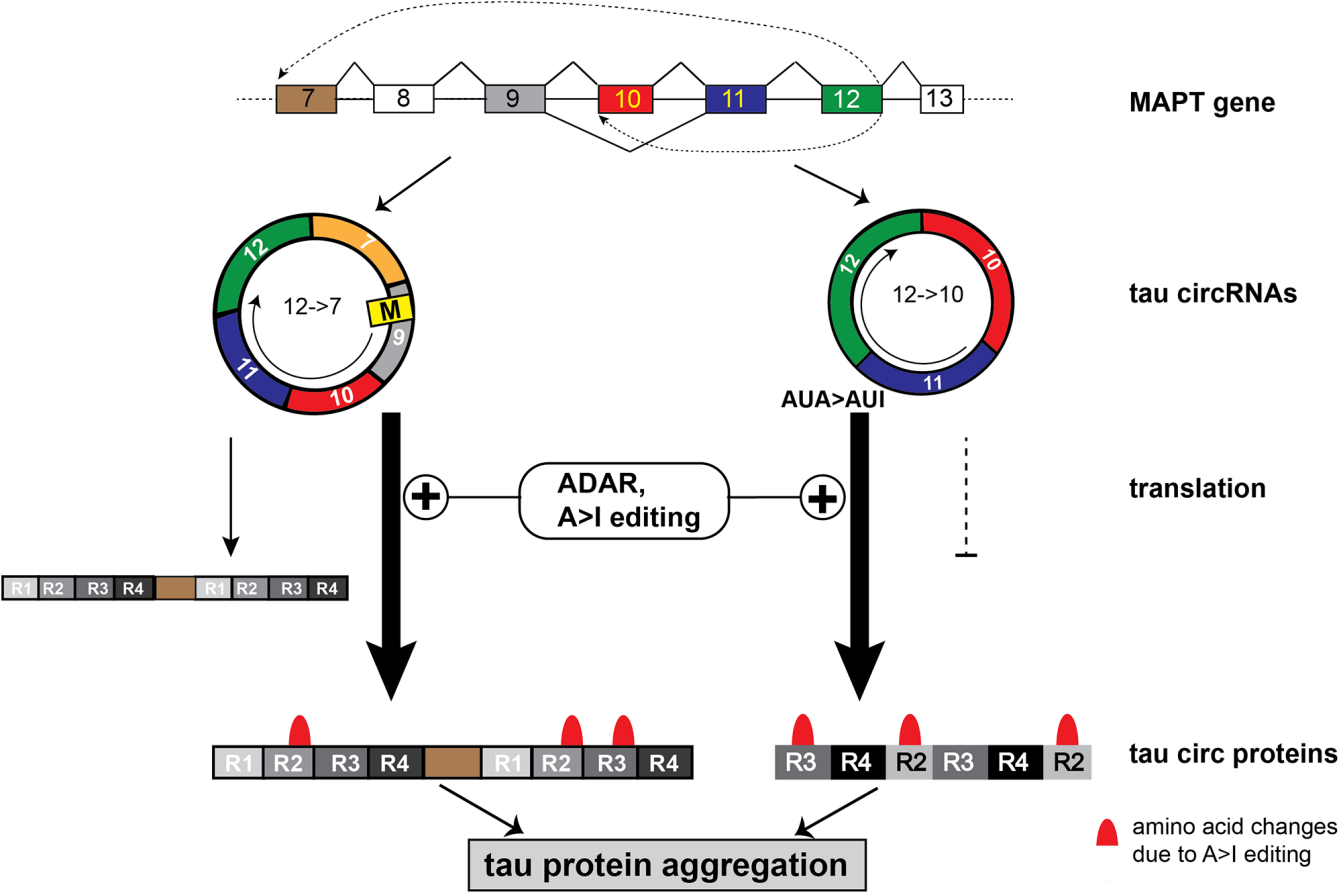
Model. The human *MAPT* gene generates two circRNAs. The 12→7 tau circRNA is translated at a low level into a protein consisting of microtubule-binding domains (thin arrow). The 12→10 tau circRNA is not translated (dotted line). ADAR-dependent A>I RNA editing strongly promotes translation of the tau circRNAs (thick arrows), including translation of the 12→10 tau circRNA due to generation of an AUI start codon. In addition, numerous mutations are introduced due to A>I editing. These proteins promote tau protein aggregation that could result in NFT formation in AD.

ADAR activity causes translation of the 12→10 tau circRNA that initially lacks a start codon. This could be significant, as this circRNA is ∼10-fold more abundant in human brain than the 12→7 circRNA. Thus, activation of ADAR enzymatic activity, possibly due to inflammatory signals (59,60), could trigger abnormal protein production from the 12→10 tau circRNA and also increase the low levels of translation of the 12→7 tau circRNA (Figure 9).

*MAPT* circRNAs are of low abundance and can be only detected using RT–PCR. The analysis of their editing status will require affinity purification of tau circRNAs. Sequencing of RNA from human brains with AD identified several highly expressed circRNAs. When taken together, the expression of 10 of these circRNAs predict clinical dementia in human post-mortem samples (61). Our data suggest that these circRNAs could be translated after A>I editing and could act as proteins.

NFTs are the pathological lesion that is most strongly associated with cognitive status in AD (4). As we observed a substantial increase in tau proteinaceous tangle formation in reporter cells exposed to tau circRNAs, this mechanism could contribute to NFT formation in the human brain, once ADAR activity increases. However, this model remains to be tested in brain, as currently the physiological circ tau protein levels are unknown. Since ADAR enzymes change multiple amino acids in the tau circRNA, the resulting proteins are likely to be a mixture of variant proteins, some resembling FTLD-Tau mutants. We propose that this aberrant mix of circ tau proteins triggers NFT formation, analogous to a less ‘penetrant’ form of FTLD-Tau. This model implies that modifying ADAR activity and/or removing tau circRNAs by targeting the backsplice sites or editing sites could be therapeutic approaches for AD and related tauopathies.

## DATA AVAILABILITY

Sequencing data have been deposited in the GEO (https://www.ncbi.nlm.nih.gov/geo/) under accession number GSE216791. Key plasmids have been deposited to Addgene (www.addgene.org), accession numbers 194161, 194163 - 194171.

## Supplementary Material

gkac1129_Supplemental_File
