# Supplementary material for: RNA editing of microtubule-associated protein tau circular RNAs promotes their translation and tau tangle formation
Source: Nucleic Acids Res. 2022 Dec 19;50(22):12979–96. doi: 10.1093/nar/gkac1129 (PMC9825173; doi:10.1093/nar/gkac1129)
Supplement: gkac1129_Supplemental_File [file gkac1129_supplemental_file.pdf]

Supplemental Figures

Supplemental Figure 1

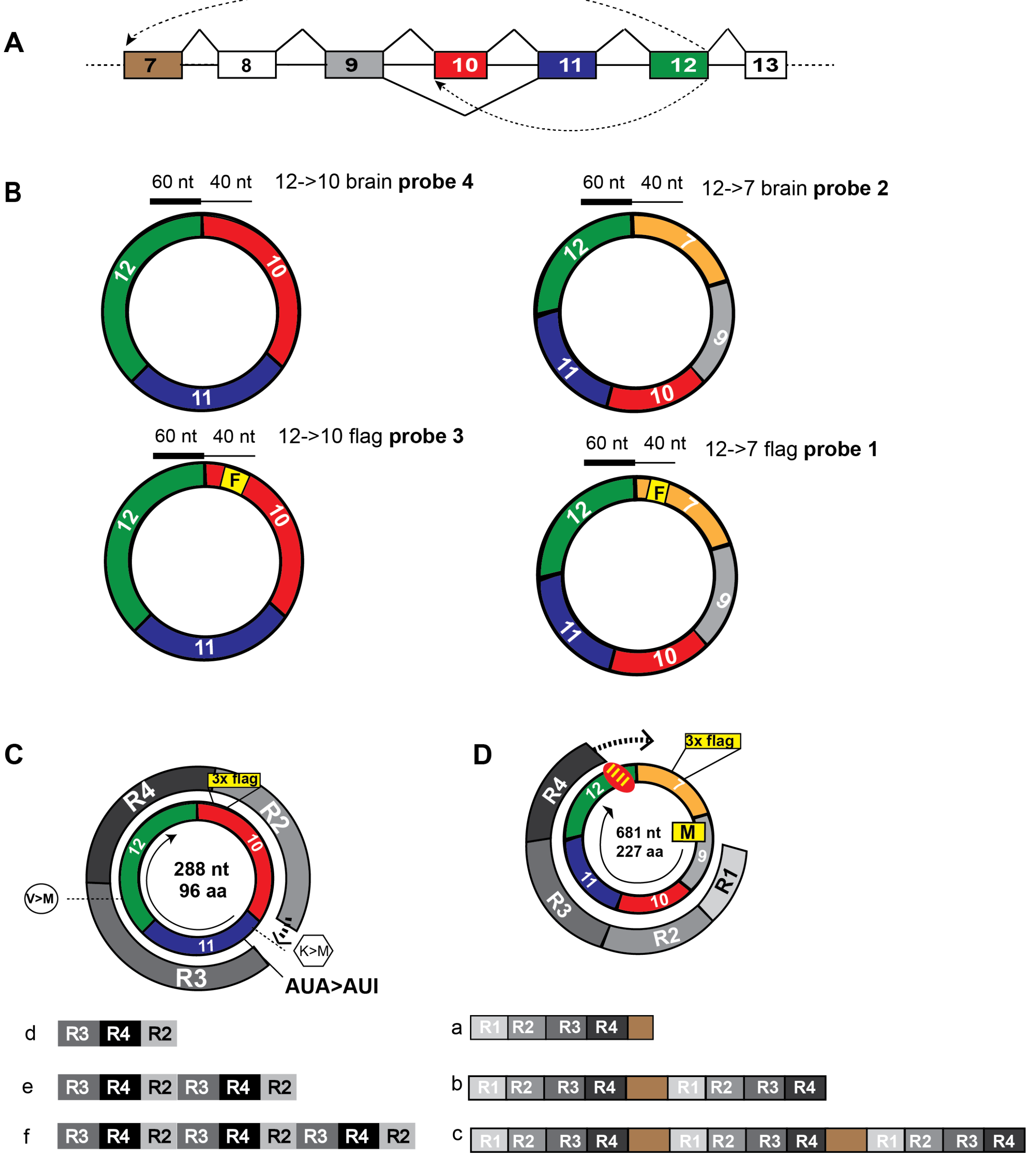

Supplemental Figure 1: RPA design and tau circRNA protein products
A. Partial structure of the MAPT gene. The backsplicing pathways 12->10 and 12->7 and the alternative splicing of exon 10 are indicated.
B. Location of the RNase protection probes across the junction sides. Probe numbers refer to Figure 1 D-E.

C. Proposed translation of the 12->10 circular RNA. The exons are indicated by coloring in the inner ring. The 3x Flag tag is shown as a yellow box. The location of the V337M and K317M mutants and the AUA that serves as a start codon after editing are indicated. The encoded protein domains are indicated as an outer ring, R2-R4: microtubule binding domains. The predicted protein products from the circular RNAs are shown underneath, d-f refer to the Western blots in Figures 2 and 3.
D. Proposed translation of the 12->7 circular RNA. The exons are indicated by coloring. The 3x Flag tag is shown as a yellow box. The predicted protein product is indicated by an outer circle. M: endogenous start codon; IIII cluster of inosines.
The predicted protein products from the circular RNAs are shown below, R1 – R4 are the MAPT binding repeats; a-c refer to the blots in Figures 2 and 3.

Supplemental Figure 2

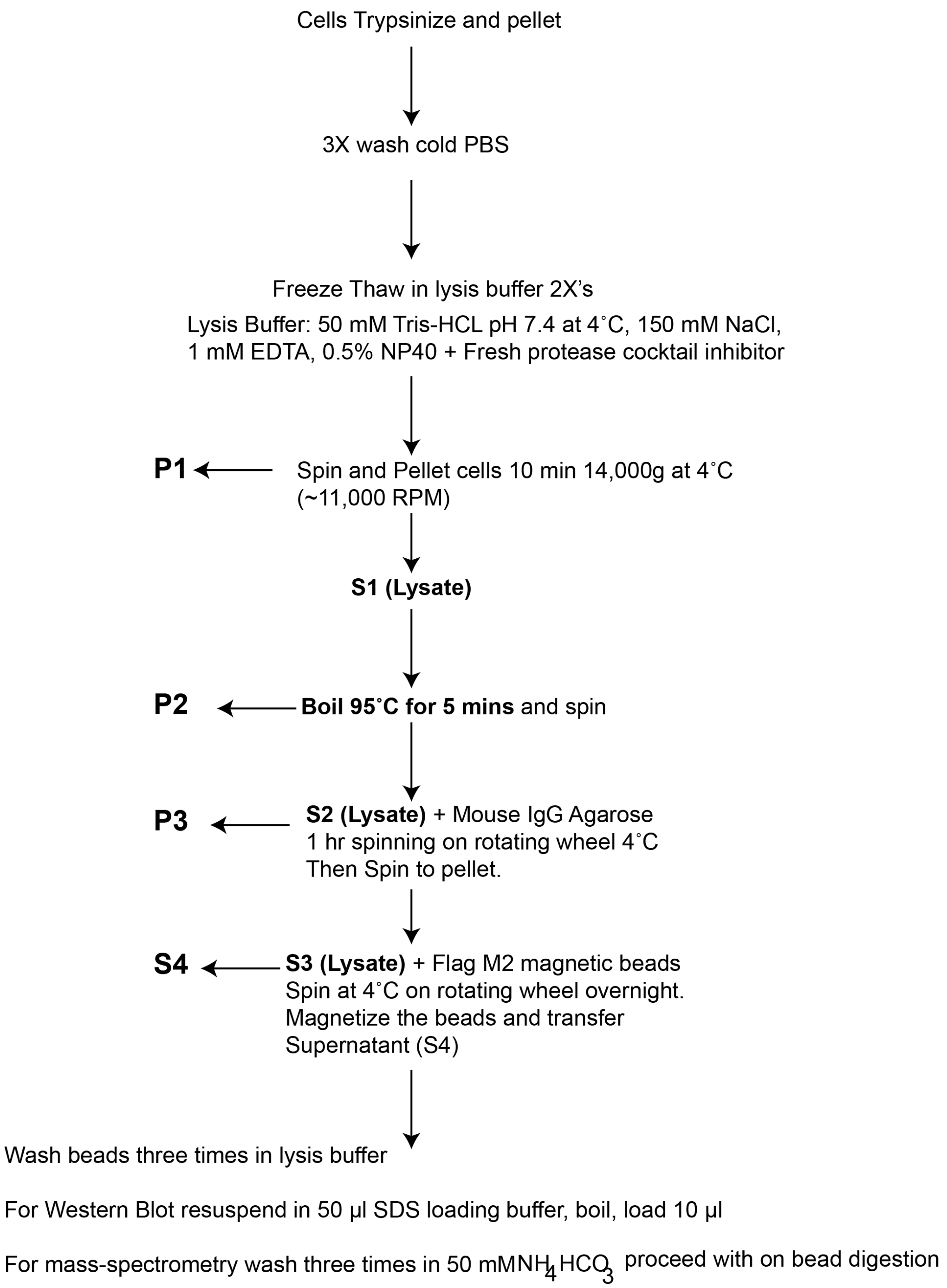

Supplemental Figure 2: Protein purification
Purification scheme of circ tau proteins after cell transfection that was used for immunoprecipitation.

Supplemental Figure 3

A

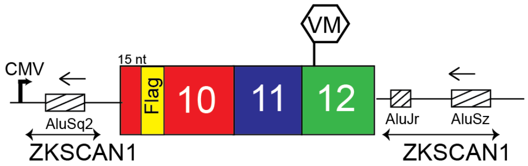

B

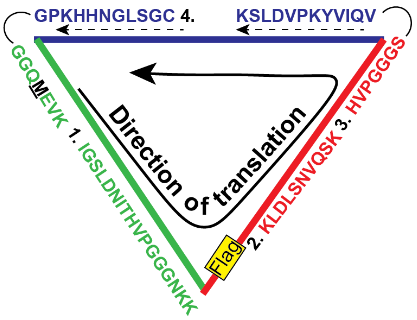

C

Peptide across exon junction

Peptides identified by mass spectromerty after tryptic digest

1. IGSLDNITHVPGGGNKK
   IGSLDNITHVPGGGN
   IGSLDNITHVPGGGNK
2. KLDLSNVQSK
   LDLSNVQSK
3. HVPGGGSVQIVY
   HVPGGGSVQIVYKPVDLSK
4. CGSLGNHHKPGGGQ<u>M</u>EVK

D **Backsplice junction after Asp-N protease digestion**

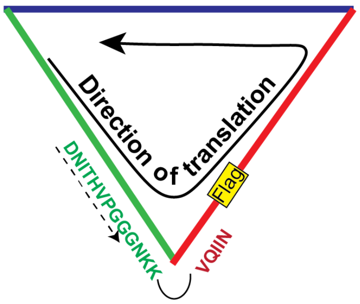

5. DNITHVPGGGNKKVQIIN

Supplemental Figure 3: Mass-spec analysis of the 12->10 V337M circ tau protein
A. Structure of the transfected construct
B. Identified peptides of the tau circRNA
C. List of peptides. The color of the peptides corresponds to the exons indicated on top.
D. The immunoprecipitated V337M circ tau protein was digested with Asp-N, which identified the junction peptide #5.

Supplemental Figure 4

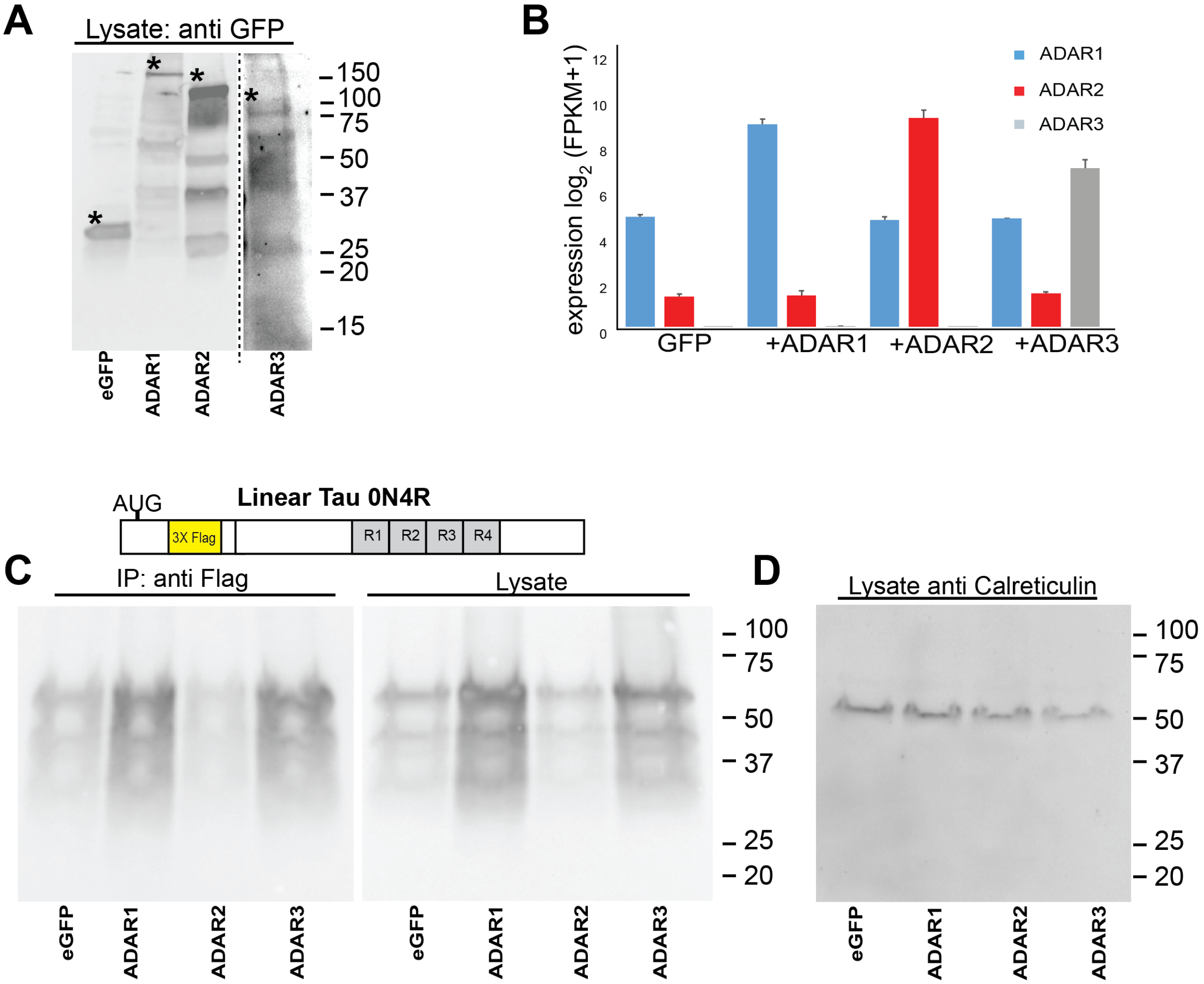

A. 5 µg of GFP tagged expression constructs for ADAR1, ADAR2 and ADAR3 were transfected into HEK293T cells and detected by Western blot. The exposure time for GFP-ADAR3 was 5x the other exposure times. The bands of expected sizes are marked with stars and are GFP: 27 kDa; GFP-ADAR1: 150 kDa; GFP-ADAR2: 90 kDa, GFP-ADAR3: 80 kDa.

B. RNA from the experiment in (A) was analyzed using RNAseq and fragments corresponding to ADAR1, 2 and 3 were determined, normalized to total transcripts.

C. An CMV-driven, N-terminal Flag tagged construct of 0N4R MAPT was cotransfected with GFP, ADAR1, ADAR2 and ADAR3 and protein expression in boiled lysates was detected by Western blot using an anti-Flag antibody.

D. The same lysates were analyzed with an antibody against calreticulin, which is heat stable.

Supplemental Figure 5

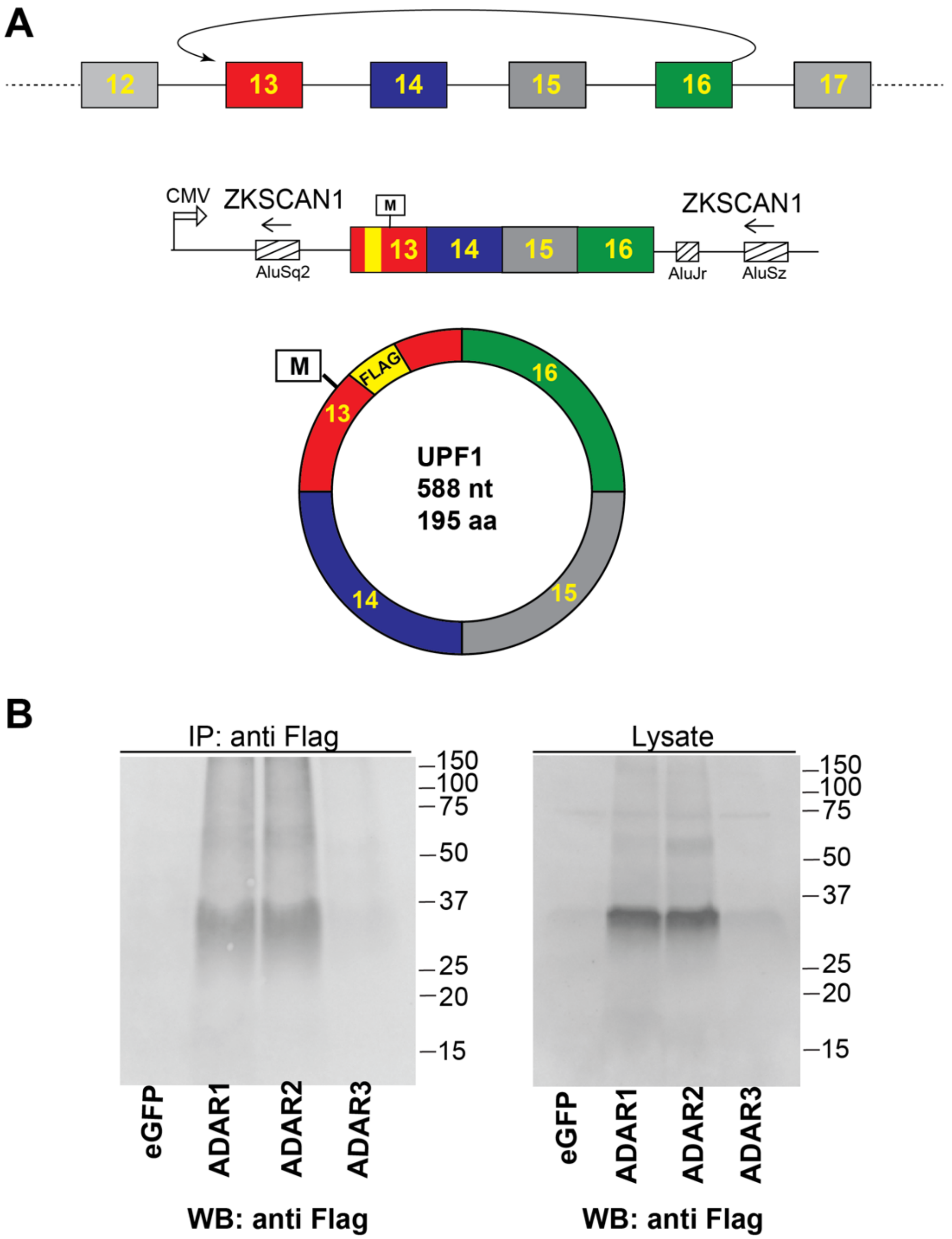

Supplemental Figure 5: Effect of ADAR1 on circ UPF1

A. Schematic diagram of the UPF1 gene region giving rise to a circRNA through backsplicing of exon 16 to 13. The expression construct is shown below, containing exons 13-16 without the UPF1 introns, flanked by ZKSCAN1 introns. The predicted circular RNA is shown below.

B. The circUPF1 expression plasmid was co-transfected into HEK293T cells with either GFP, ADAR1, ADAR2, and ADAR3 expression clones as indicated. The protein was immunoprecipitated using native salt conditions and was detected using anti Flag antibody.

Supplemental Figure 6: Quantification of A>I RNA editing of the tau circRNA

Pos: position in the 12->10 and 12->7 tau circRNAs.
The first nucleotide in exon 10 is position #1 for the 12->10 tau circRNA
The first nucleotide in exon 10 is position #409 for the 12->7 tau circRNA

GFP, ADAR1, ADAR2, ADAR3: per cent A>G change after co-transfection with 1 µg of the respective expression constructs

A. Comparison of the residues for exons 10 to 12

	Flanked by ZKSCAN1 alu 12->10					Flanked by tau alu 12->10					Flanked by tau alu 12->7			
POS	GFP	ADAR1	ADAR2	ADAR3	POS	GFP	ADAR1	ADAR2	ADAR3	POS	GFP	ADAR1	ADAR 2	ADAR 3
1	0	0	0	0	1	0	0	0	0	409	0	0	0	0
2	0	0	0	0	2	0	0	0	0	410	0	0	0	0
3	0	0	0	0	3	0	0	0	0	411	0	0	0	0
4	0	0	0	0	4	0	0	0	0	412	0	0	0	0
5	0	0	0	0	5	0	0	0	0	413	0	0	0	0
6	0	0	0	0	6	0	0	0	0	414	0	0	0	0
7	0	0	0	0	7	0	0	0	0	415	0	0	0	0
8	0	0	0	0	8	0	0	0	0	416	0	0	0	0
9	0	0	0	0	9	0	0	0	0	417	0	0	0	0
10	0	0	0	0	10	0	0	0	0	418	0	0	0	0
11	0	0	0	0	11	0	0	0	0	419	0	0	0	0
12	0	0	0	0	12	0	0	0	0	420	0	0	0	0
13	0	0	0	0	13	0	0	0	0	421	0	0	0	0
14	0	0	0	0	14	0	0	0	0	422	0	0	0	0
15	0	0	0	0	15	0	0	0	0	423	0	0	0	0
16	0	0	0	0	16	0	0	0	0	424	0	0	0	0
17	0.71%	1.38%	0.93%	0.61%	17	0.00%	1.31%	1.10%	0.00%	425	0	0	0	0
18	0	0	0	0	18	0	0	0	0	426	0	0	0	0
19	0	0	0	0	19	0	0	0	0	427	0	0	0	0
20	1.21%	1.69%	0.53%	0.44%	20	0.00%	0.68%	1.23%	0.80%	428	0.00%	4.76%	4.35%	0.00%
21	0	0	0	0	21	0	0	0	0	429	0	0	0	0
22	0	0	0	0	22	0	0	0	0	430	0	0	0	0
23	0	0	0	0	23	0	0	0	0	431	0	0	0	0
24	0	0	0	0	24	0	0	0	0	432	0	0	0	0
25	0	0	0	0	25	0	0	0	0	433	0	0	0	0
26	0.08%	0.08%	0.00%	0.00%	26	0.00%	0	1.43%	0.00%	434	0	0	0	0
27	0	0	0	0	27	0	0	0	0	435	0	0	0	0
28	0	0	0	0	28	0	0	0	0	436	0	0	0	0
29	0	0	0	0	29	0	0	0	0	437	0	0	0	0
30	0	0	0	0	30	0	0	0	0	438	0	0	0	0
31	3.65%	3.96%	0.93%	2.05%	31	0.00%	3.33%	2.86%	1.94%	439	0.00%	4.55%	4.17%	0.00%
32	0	0	0	0	32	0	0	0	0	440	0	0	0	0
33	0	0	0	0	33	0	0	0	0	441	0	0	0	0
34	0.87%	1.89%	0.37%	0.96%	34	0.00%	0.00%	1.39%	0.00%	442	0	0	0	0
35	1.41%	2.04%	1.12%	0.69%	35	0.00%	1.65%	0	0.00%	443	0.00%	4.55%	4.17%	0.00%
36	0	0	0	0	36	0	0	0	0	444	0	0	0	0
37	0	0	0	0	37	0	0	0	0	445	0	0	0	0
38	0	0	0	0	38	0	0	0	0	446	0	0	0	0
39	0	0	0	0	39	0	0	0	0	447	0	0	0	0
40	0	0	0	0	40	0	0	0	0	448	0	0	0	0
41	0	0	0	0	41	0	0	0	0	449	0	0	0	0
42	0	0	0	0	42	0	0	0	0	450	0	0	0	0
43	0	0	0	0	43	0	0	0	0	451	0	0	0	0

44	0	0	0	0	44	0	0	0	0	452	0	0	0	0
45	0	0	0	0	45	0	0	0	0	453	0	0	0	0
46	0	0	0	0	46	0	0	0	0	454	0	0	0	0
47	0.85%	1.10%	0.65%	0.58%	47	0.00%	0.00%	0.00%	0.00%	455	0	0	0	0
48	0	0	0	0	48	0	0	0	0	456	0	0	0	0
49	0	0	0	0	49	0	0	0	0	457	0	0	0	0
50	0	0	0	0	50	0	0	0	0	458	0	0	0	0
51	0	0	0	0	51	0	0	0	0	459	0	0	0	0
52	0	0	0	0	52	0	0	0	0	460	0	0	0	0
53	0	0	0	0	53	0	0	0	0	461	0	0	0	0
54	0	0	0	0	54	0	0	0	0	462	0	0	0	0
55	0	0	0	0	55	0	0	0	0	463	0	0	0	0
56	0	0	0	0	56	0	0	0	0	464	0	0	0	0
57	0.50%	0.84%	0.67%	0.00%	57	0.00%	2.06%	0.00%	0.00%	465	0	0	0	0
58	0.79%	1.40%	0.45%	1.04%	58	0.00%	0.00%	0.00%	1.20%	466	0	0	0	0
59	0.84%	1.81%	1.19%	2.60%	59	0.00%	0.00%	0.00%	0.00%	467	0	0	0	2.86%
60	0	0	0	0	60	0	0	0	0	468	0	0	0	0
61	0	0	0	0	61	0	0	0	0	469	0	0	0	0
62	0	0	0	0	62	0	0	0	0	470	0	0	0	0
63	0	0	0	0	63	0	0	0	0	471	0	0	0	0
64	1.22%	1.95%	0.93%	1.21%	64	0.00%	1.19%	0.00%	0.00%	472	0.00%	2.78%	2.63%	0.00%
65	2.01%	2.43%	1.63%	2.23%	65	0.00%	1.18%	0.00%	0.00%	473	0.00%	2.78%	2.63%	0.00%
66	0	0	0	0	66	0	0	0	0	474	0	0	0	0
67	1.86%	2.18%	1.00%	2.14%	67	0.00%	1.16%	0.00%	0.00%	475	0.00%	2.63%	2.50%	0.00%
68	0	0	0	0	68	0	0	0	0	476	0	0	0	0
69	0	0	0	0	69	0	0	0	0	477	0	0	0	0
70	1.49%	2.30%	0.42%	1.39%	70	0.00%	1.18%	0.00%	0.00%	478	0.00%	2.33%	2.22%	0.00%
71	1.63%	2.36%	0.93%	0.99%	71	0.00%	2.35%	0.00%	0.00%	479	0.00%	0.00%	0.00%	2.04%
72	1.64%	2.60%	1.18%	1.39%	72	0.00%	0.00%	0.00%	2.86%	480	0.00%	2.38%	2.27%	2.04%
73	0	0	0	0	73	0	0	0	0	481	0	0	0	0
74	0	0	0	0	74	0	0	0	0	482	0	0	0	0
75	0	0	0	0	75	0	0	0	0	483	0	0	0	0
76	0	0	0	0	76	0	0	0	0	484	0	0	0	0
77	0	0	0	0	77	0	0	0	0	485	0	0	0	0
78	0	0	0	0	78	0	0	0	0	486	0	0	0	0
79	0	0	0	0	79	0	0	0	0	487	0	0	0	0
80	0	0	0	0	80	0	0	0	0	488	0	0	0	0
81	0	0	0	0	81	0	0	0	0	489	0	0	0	0
82	0	0	0	0	82	0	0	0	0	490	0	0	0	0
83	0	0	0	0	83	0	0	0	0	491	0	0	0	0
84	0	0	0	0	84	0	0	0	0	492	0	0	0	0
85	0	0	0	0	85	0	0	0	0	493	0	0	0	0
86	0	0	0	0	86	0	0	0	0	494	0	0	0	0
87	0	0	0	0	87	0	0	0	0	495	0	0	0	0
88	0	0	0	0	88	0	0	0	0	496	0	0	0	0
89	0	0	0	0	89	0	0	0	0	497	0	0	0	0
90	0	0	0	0	90	0	0	0	0	498	0	0	0	0
91	0.15%	0.53%	0.53%	0.00%	91	0.00%	0.00%	2.00%	0.00%	499	0.00%	1.75%	1.67%	0.00%
92	0	0	0	0	92	0	0	0	0	500	0	0	0	0
93	0	0	0	0	93	0	0	0	0	501	0	0	0	0
94	0	0	0	0	94	0	0	0	0	502	0	0	0	0
95	0	0	0	0	95	0	0	0	0	503	0	0	0	0
96	0	0	0	0	96	0	0	0	0	504	0	0	0	0
97	0	0	0	0	97	0	0	0	0	505	0	0	0	0
98	0.39%	0.69%	0.18%	0.21%	98	0.00%	1.25%	1.96%	0.00%	506	0.00%	1.64%	1.56%	0.00%
99	1.14%	2.90%	0.88%	1.25%	99	0.00%	1.28%	2.00%	1.67%	507	0.00%	3.28%	3.13%	1.25%
100	1.08%	2.32%	0.87%	0.63%	100	0.00%	1.27%	2.00%	0.00%	508	0	0	0	0
101	0	0	0	0	101	0	0	0	0	509	0	0	0	0
102	1.72%	2.84%	2.18%	2.09%	102	0.00%	0.00%	2.00%	1.67%	510	0.00%	1.64%	1.56%	1.25%
103	0	0	0	0	103	0	0	0	0	511	0	0	0	0
104	0	0	0	0	104	0	0	0	0	512	0	0	0	0
105	0	0	0	0	105	0	0	0	0	513	0	0	0	0
106	0	0	0	0	106	0	0	0	0	514	0	0	0	0
107	1.32%	2.95%	1.09%	1.20%	107	0.00%	1.25%	2.08%	0.00%	515	0.00%	3.28%	3.13%	1.18%
108	0	0	0	0	108	0	0	0	0	516	0	0	0	0
109	0	0	0	0	109	0	0	0	0	517	0	0	0	0
110	1.03%	2.52%	0.58%	0.39%	110	0.00%	1.32%	0.00%	0.00%	518	0.00%	0.00%	0.00%	1.18%

111	1.13%	2.57%	0.74%	0.58%	111	0.00%	1.33%	0.00%	0.00%	519	0.00%	1.69%	1.61%	1.15%
112	0	0	0	0	112	0	0	0	0	520	0	0	0	0
113	0	0	0	0	113	0	0	0	0	521	0	0	0	0
114	0.10%	0.39%	2.07%	0.39%	114	0.00%	1.35%	2.04%	0.00%	522	0.00%	1.67%	1.59%	0.00%
115	0	0	0	0	115	0	0	0	0	523	0	0	0	0
116	0	0	0	0	116	0	0	0	0	524	0	0	0	0
117	0	0	0	0	117	0	0	0	0	525	0	0	0	0
118	0	0	0	0	118	0	0	0	0	526	0	0	0	0
119	0.31%	0.46%	0.42%	0.00%	119	0.00%	0.00%	2.04%	0.00%	527	0	0	0	0
120	0	0	0	0	120	0	0	0	0	528	0	0	0	0
121	0	0	0	0	121	0	0	0	0	529	0	0	0	0
122	0	0	0	0	122	0	0	0	0	530	0	0	0	0
123	0	0	0	0	123	0	0	0	0	531	0	0	0	0
124	0.19%	0.86%	0.30%	0.18%	124	0.00%	0.00%	1.75%	0.00%	532	0	0	0	0
125	0	0	0	0	125	0	0	0	0	533	0	0	0	0
126	0	0	0	0	126	0	0	0	0	534	0	0	0	0
127	0.54%	2.02%	0.69%	0.18%	127	0.00%	1.28%	1.75%	0.00%	535	0	0	0	0
128	1.02%	2.61%	1.67%	0.73%	128	0.00%	1.23%	3.33%	0.00%	536	0	1.64%	1.54%	1.83%
129	0	0	0	0	129	0	0	0	0	537	0	0	0	0
130	0	0	0	0	130	0	0	0	0	538	0	0	0	0
131	0	0	0	0	131	0	0	0	0	539	0	0	0	0
132	0	0	0	0	132	0	0	0	0	540	0	0	0	0
133	0.43%	1.27%	0.52%	0.53%	133	0.00%	1.12%	3.28%	0.00%	541	0	1.61%	1.54%	0.83%
134	0	0	0	0	134	0	0	0	0	542	0	0	0	0
135	0	0	0	0	135	0	0	0	0	543	0	0	0	0
136	0	0	0	0	136	0	0	0	0	544	0	0	0	0
137	0	0	0	0	137	0	0	0	0	545	0	0	0	0
138	0	0	0	0	138	0	0	0	0	546	0	0	0	0
139	0.24%	0.56%	0.35%	0.17%	139	0.00%	1.04%	1.49%	0.00%	547	0	1.49%	1.43%	0
140	0.62%	2.33%	1.13%	0.86%	140	0.00%	1.04%	2.90%	0.00%	548	0	1.49%	1.43%	0.79%
141	0	0	0	0	141	0	0	0	0	549	0	0	0	0
142	0	0	0	0	142	0	0	0	0	550	0	0	0	0
143	0	0	0	0	143	0	0	0	0	551	0	0	0	0
144	0	0	0	0	144	0	0	0	0	552	0	0	0	0
145	0	0	0	0	145	0	0	0	0	553	0	0	0	0
146	0	0	0	0	146	0	0	0	0	554	0	0	0	0
147	0	0	0	0	147	0	0	0	0	555	0	0	0	0
148	0	0	0	0	148	0	0	0	0	556	0	0	0	0
149	0	0	0	0	149	0	0	0	0	557	0	0	0	0
150	0.14%	0.28%	0.86%	0.00%	150	0.00%	1.01%	1.41%	0.00%	558	0.00%	1.39%	1.32%	0
151	0	0	0	0	151	0	0	0	0	559	0	0	0	0
152	0	0	0	0	152	0	0	0	0	560	0	0	0	0
153	3.48%	3.90%	1.65%	1.24%	153	0.00%	2.02%	16.22%	0.00%	561	0.00%	1.39%	1.33%	1.37%
154	0	0	0	0	154	0	0	0	0	562	0	0	0	0
155	0	0	0	0	155	0	0	0	0	563	0	0	0	0
156	0	0	0	0	156	0	0	0	0	564	0	0	0	0
157	0	0	0	0	157	0	0	0	0	565	0	0	0	0
158	1.20%	2.54%	1.20%	0.63%	158	0.00%	1.04%	5.19%	0.00%	566	0.00%	1.28%	1.23%	1.30%
159	0	0	0	0	159	0	0	0	0	567	0	0	0	0
160	0.46%	0.79%	0.26%	0.46%	160	0.00%	1.94%	0.00%	0.00%	568	0	0	0	0
161	0	0	0	0	161	0	0	0	0	569	0	0	0	0
162	0	0	0	0	162	0	0	0	0	570	0	0	0	0
163	0	0	0	0	163	0	0	0	0	571	0	0	0	0
164	0	0	0	0	164	0	0	0	0	572	0	0	0	0
165	0	0	0	0	165	0	0	0	0	573	0	0	0	0
166	0	0	0	0	166	0	0	0	0	574	0	0	0	0
167	0.69%	1.16%	0.29%	0.00%	167	0.00%	1.64%	1.18%	0.00%	575	0	0	0	0
168	0	0	0	0	168	0	0	0	0	576	0	0	0	0
169	1.51%	2.31%	0.80%	0.52%	169	0.00%	2.42%	0.00%	0.00%	577	0.00%	1.06%	0.85%	0.53%
170	0.83%	1.88%	0.79%	0.00%	170	6.25%	0.79%	0.00%	0.00%	578	0.00%	1.04%	0.85%	0.53%
171	0.08%	0.21%	0.97%	0.13%	171	6.25%	0.00%	1.12%	0.00%	579	0.00%	0.98%	0.81%	0.00%
172	0	0	0	0	172	0	0	0	0	580	0	0	0	0
173	0	0	0	0	173	0	0	0	0	581	0	0	0	0
174	0.21%	0.53%	1.21%	0.00%	174	0.00%	0.00%	4.08%	0.00%	582	0.00%	0.88%	0.76%	0.00%
175	0	0	0	0	175	0	0	0	0	583	0	0	0	0
176	0	0	0	0	176	0	0	0	0	584	0	0	0	0
177	0	0	0	0	177	0	0	0	0	585	0.00%	0.87%	0.75%	0.00%

178	0	0	0	0	178	0	0	0	0	586	0	0	0	0
179	0	0	0	0	179	0	0	0	0	587	0	0	0	0
180	0	0	0	0	180	0	0	0	0	588	0	0	0	0
181	0	0	0	0	181	0	0	0	0	589	0	0	0	0
182	0	0	0	0	182	0	0	0	0	590	0	0	0	0
183	0	0	0	0	183	0	0	0	0	591	0	0	0	0
184	0	0	0	0	184	0	0	0	0	592	0	0	0	0
185	0.17%	0.50%	1.29%	0.00%	185	0.00%	0.00%	0.95%	0.00%	593	0	0	0	0
186	0	0	0	0	186	0	0	0	0	594	0	0	0	0
187	0	0	0	0	187	0	0	0	0	595	0	0	0	0
188	0	0	0	0	188	0	0	0	0	596	0	0	0	0
189	0	0	0	0	189	0	0	0	0	597	0	0	0	0
190	0	0	0	0	190	0	0	0	0	598	0	0	0	0
191	0.03%	0.04%	0.05%	0.00%	191	7.69%	0.00%	0.00%	0.00%	599	0	0	0	0
192	0.47%	1.17%	1.05%	0.40%	192	0.00%	0.61%	0.00%	0.00%	600	3.13%	0.76%	0.65%	0.42%
193	0	0	0	0	193	0	0	0	0	601	0	0	0	0
194	0	0	0	0	194	0	0	0	0	602	0	0	0	0
195	0.94%	2.04%	1.62%	0.69%	195	4.00%	2.35%	4.63%	0.00%	603	0.00%	0.70%	0.63%	0.40%
196	0.71%	1.87%	1.67%	0.30%	196	4.00%	0.59%	2.78%	0.00%	604	0	0	0	0.40%
197	0.47%	0.77%	0.63%	0.49%	197	0.00%	0.59%	1.87%	0.00%	605	0	0	0	0
198	1.19%	1.96%	1.14%	0.20%	198	0.00%	1.18%	0.00%	0.00%	606	0.00%	1.44%	1.27%	0
199	0	0	0	0	199	0	0	0	0	607	0	0	0	0
200	0	0	0	0	200	0	0	0	0	608	0	0	0	0
201	0	0	0	0	201	0	0	0	0	609	0	0	0	0
202	0	0	0	0	202	0	0	0	0	610	0	0	0	0
203	0.45%	1.63%	0.73%	0.60%	203	4.17%	1.78%	0.00%	0.00%	611	0	0	0	0
204	0	0	0	0	204	0	0	0	0	612	0	0	0	0
205	0	0	0	0	205	0	0	0	0	613	0	0	0	0
206	1.25%	3.03%	1.80%	0.49%	206	0.00%	1.16%	3.60%	0.00%	614	0.00%	0.72%	0.64%	0
207	0	0	0	0	207	0	0	0	0	615	0	0	0	0
208	0	0	0	0	208	0	0	0	0	616	0	0	0	0
209	0	0	0	0	209	0	0	0	0	617	0	0	0	0
210	0	0	0	0	210	0	0	0	0	618	0	0	0	0
211	0	0	0	0	211	0	0	0	0	619	0	0	0	0
212	0	0	0	0	212	0	0	0	0	620	0	0	0	0
213	0	0	0	0	213	0	0	0	0	621	0	0	0	0
214	0	0	0	0	214	0	0	0	0	622	0	0	0	0
215	0	0	0	0	215	0	0	0	0	623	0	0	0	0
216	0	0	0	0	216	0	0	0	0	624	0	0	0	0
217	0.33%	1.47%	0.57%	0.09%	217	0.00%	1.09%	0.00%	0.00%	625	0	0	0	0
218	0.57%	3.29%	1.55%	0.29%	218	3.45%	1.09%	2.59%	0.00%	626	0.00%	0.75%	0.66%	0.00%
219	0	0	0	0	219	0	0	0	0	627	0	0	0	0
220	0	0	0	0	220	0	0	0	0	628	0	0	0	0
221	0	0	0	0	221	0	0	0	0	629	0	0	0	0
222	0	0	0	0	222	0	0	0	0	630	0	0	0	0
223	0.90%	2.75%	1.08%	0.46%	223	0.00%	1.02%	0.00%	0.00%	631	0.00%	0.74%	0.64%	0.39%
224	0	0	0	0	224	0	0	0	0	632	0	0	0	0
225	0.13%	0.96%	0.45%	0.00%	225	0.00%	0.99%	3.28%	0.00%	633	0.00%	0.00%	0.00%	0.78%
226	0	0	0	0	226	0	0	0	0	634	0	0	0	0
227	0	0	0	0	227	0	0	0	0	635	0	0	0	0
228	0	0	0	0	228	0	0	0	0	636	0	0	0	0
229	0	0	0	0	229	0	0	0	0	637	0	0	0	0
230	0.44%	1.06%	0.63%	0.00%	230	0.00%	0.40%	0.00%	0.00%	638	0.00%	1.45%	1.86%	0.00%
231	0	0	0	0	231	0	0	0	0	639	0	0	0	0
232	0	0	0	0	232	0	0	0	0	640	0	0	0	0
233	0	0	0	0	233	0	0	0	0	641	0	0	0	0
234	0	0	0	0	234	0	0	0	0	642	0	0	0	0
235	0	0	0	0	235	0	0	0	0	643	0	0	0	0
236	1.52%	2.79%	0.60%	0.84%	236	0.00%	2.02%	0.00%	0.00%	644	0.00%	1.47%	1.90%	0.40%
237	0	0	0	0	237	0	0	0	0	645	0	0	0	0
238	0	0	0	0	238	0	0	0	0	646	0	0	0	0
239	0	0	0	0	239	0	0	0	0	647	0	0	0	0
240	0	0	0	0	240	0	0	0	0	648	0	0	0	0
241	0	0	0	0	241	0	0	0	0	649	0	0	0	0
242	0	0	0	0	242	0	0	0	0	650	0	0	0	0
243	0	0	0	0	243	0	0	0	0	651	0	0	0	0
244	0	0	0	0	244	0	0	0	0	652	0	0	0	0

245	0	0	0	0	245	0	0	0	0	653	0	0	0	0
246	0	0	0	0	246	0	0	0	0	654	0	0	0	0
247	0	0	0	0	247	0	0	0	0	655	0	0	0	0
248	0	0	0	0	248	0	0	0	0	656	0	0	0	0
249	0	0	0	0	249	0	0	0	0	657	0	0	0	0
250	0	0	0	0	250	0	0	0	0	658	0	0	0	0
251	0	0	0	0	251	0	0	0	0	659	0	0	0	0
252	0	0	0	0	252	0	0	0	0	660	0	0	0	0
253	0	0	0	0	253	0	0	0	0	661	2.99%	0.00%	0.00%	0.00%
254	0.54%	1.22%	0.65%	0.15%	254	0.00%	0.83%	0.00%	0.00%	662	0.00%	0.45%	0.34%	0.00%
255	0	0	0	0	255	0	0	0	0	663	0	0	0	0
256	0.51%	1.14%	0.40%	0.15%	256	0.00%	0.83%	0.00%	1.68%	664	0.00%	0.45%	0.34%	0.00%
257	0	0	0	0	257	0	0	0	0	665	0	0	0	0
258	0	0	0	0	258	0	0	0	0	666	0	0	0	0
259	0	0	0	0	259	0	0	0	0	667	0.00%	0.44%	0.34%	0.00%
260	0	0	0	0	260	0	0	0	0	668	0	0	0	0
261	0	0	0	0	261	0	0	0	0	669	0	0	0	0
262	0	0	0	0	262	0	0	0	0	670	0	0	0	0
263	0.03%	0.25%	0.46%	0.00%	263	0.00%	0.00%	1.88%	0.00%	671	0.00%	0.44%	1.00%	0.00%
264	0	0	0	0	264	0	0	0	0	672	0	0	0	0
265	0	0	0	0	265	0	0	0	0	673	0	0	0	0
266	0	0	0	0	266	0	0	0	0	674	0	0	0	0
267	0	0	0	0	267	0	0	0	0	675	0	0	0	0
268	0	0	0	0	268	0	0	0	0	676	0	0	0	0
269	0	0	0	0	269	0	0	0	0	677	0	0	0	0
270	0	0	0	0	270	0	0	0	0	678	0	0	0	0
271	0	0	0	0	271	0	0	0	0	679	0	0	0	0
272	0	0	0	0	272	0	0	0	0	680	0	0	0	0
273	0	0	0	0	273	0	0	0	0	681	0	0	0	0
274	0	0	0	0	274	0	0	0	0	682	0	0	0	0
275	0	0	0	0	275	0	0	0	0	683	0	0	0	0
276	0	0	0	0	276	0	0	0	0	684	0	0	0	0
277	0	0	0	0	277	0	0	0	0	685	0	0	0	0
278	0	0	0	0	278	0	0	0	0	686	0	0	0	0
279	0	0	0	0	279	0	0	0	0	687	0	0	0	0
280	0	0	0	0	280	0	0	0	0	688	0	0	0	0
281	0	0	0	0	281	0	0	0	0	689	0	0	0	0
282	0	0	0	0	282	0	0	0	0	690	0	0	0	0
283	0	0	0	0	283	0	0	0	0	691	0	0	0	0
284	0	0	0	0	284	0	0	0	0	692	0	0	0	0
285	0	0	0	0	285	0	0	0	0	693	0	0	0	0
286	0	0	0	0	286	0	0	0	0	694	0	0	0	0
287	0	0	0	0	287	0	0	0	0	695	0	0	0	0
288	0	0	0	0	288	0	0	0	0	696	0	0	0	0

B. Editing profile for Exons 7-9 of the 12->7 tau circRNA

Flanked by tau alu 12->7				
**POS**	**GFP**	**ADAR 1**	**ADAR 2**	**ADAR 3**
1	0	0	0	0
2	0	0	0	0
3	0	0	0	0
4	0	0	0	0
5	0	0	0	0
6	0	0	0	0
7	0	0	0	0
8	0	0	0	0
9	0	0	0	0
10	0	0	0	0
11	0	0	0	0
12	0	0	0	0

13	0	0	0	0
14	0	0	0	0
15	0	0	0	0
16	0	0	0	0
17	0	0	0	0
18	0	0	0	0
19	0	0	0	0
20	0	0	0	0
21	0	0	0	0
22	0	0	0	0
23	0	0	0	0
24	0	0	0	0
25	0	0	0	0
26	0	3.06%	0.79%	0
27	0	0	0	0
28	0	0	0	0
29	0	2.04%	0.79%	0
30	0	1.70%	0.79%	0
31	0	1.71%	0.80%	0
32	0	0	0	0
33	0	0	0	0
34	0	0	0	0
35	0	1.85%	0.84%	0
36	0	0	0	0
37	0	0	0	0
38	0	0	0	0
39	0	0	0	0
40	0	0	0	0
41	0	0	0	0
42	0	0	0	0
43	0	0	0	0
44	0	0	0	0
45	0	0	0	0
46	0	0	0	0
47	0	0	0	0
48	0	0	0	0
49	0	0	0	0
50	0	0	0	0
51	0	0	0	0
52	0	0	0	0
53	0	0	0	0
54	0	0	0	0
55	0	0	0	0
56	0	0	0	0
57	0	0	0	0
58	0	0	0	0
59	0	0	0	0
60	0	0	0	0
61	0	0	0	0
62	0	0	0	0
63	0	0	0	0
64	0	0	0	0
65	0	0	0	0
66	0	0	0	0
67	0	0	0	0
68	0	0	0	0
69	0	0	0	0
70	0	0	0	0
71	0	0	0	0
72	0	0	0	0
73	0	1.05%	0.	0
74	0	1.05%	0.94%	0
75	0	0	0	0
76	0	0	0	0
77	0	0	0	0
78	0	0	0	0
79	0	0	0	0

80	0	0	0	0
81	0	0	0	0
82	0	0	0	0
83	0	0	0	0
84	0	0	0	0
85	0	0	0	0
86	0	0	1.10%	0
87	0	0	0	0
88	0	0	0	0
89	0	0	0	0
90	0	0	0	0
91	0	0	0	0
92	0	0	0	0
93	0	0	0	0
94	0	0	0	0
95	0	0	0	0
96	0	0	0	0
97	0	0	0	0
98	0	0	0	0
99	0	0	0	0
100	0	0	0	0
101	0	0	0	0
102	0	0	0	0
103	0	0	0	0
104	0	0	0	0
105	0	0	0	0
106	0	1.79%	0.86%	0
107	0	0	0	0
108	0	0	0	0
109	0	0	1.72%	0
110	0	0	0	0
111	0	0	0	0
112	0	0	0	0
113	0	0	0	0
114	0	0	0	0
115	0	0	0	0
116	0	0	0	0
117	0	0	0	0
118	0	0	0	0
119	0	0	0	0
120	0	0	0	0
121	0	0	0	0
122	0	0	0	0
123	0	0	0	0
124	0	0	1.49%	0
125	0	1.35%	1.49%	0
126	0	0	0	0
127	0	0	0	0
128	0	0	0	0
129	0	1.37%	1.54%	0
130	0	0	0	0
131	0	0	0	0
132	0	0	3.23%	0
133	0	0	0	0
134	0	0	0	0
135	0	0	0	0
136	0	0	0	0
137	0	0	0	0
138	0	0	0	0
139	0	0	0	0
140	0	0	0	0
141	0	0	0	0
142	0	0	0	0
143	0	0	0	0
144	0	0	0	0
145	0	0	0	0
146	0	0	0	0

147	0	1.64%	0	0
148	0	0	0	0
149	0	0	0	0
150	0	0	0	0
151	0	0	0	0
152	0	0	0	0
153	0	0	0	0
154	0	0	0	0
155	0	1.75%	0	0
156	0	0	0	0
157	0	0	0	0
158	0	0	0	0
159	0	1.75%	3.77%	0
160	0	0	0	0
161	0	0	0	0
162	0	0	0	0
163	0	0	0	0
164	0	0	0	0
165	0	0	0	0
166	0	0	0	0
167	0	0	0	0
168	0	0	0	0
169	0	1.79%	2.13%	0
170	0	0	0	0
171	0	0	0	0
172	0	0	0	0
173	0	0	0	0
174	0	0	0	0
175	0	0	0	0
176	0	13.21%	4.35%	0
177	0	0	0	0
178	0	0	0	0
179	0	0	0	0
180	0	0	0	0
181	0	0	0	0
182	0	0	0	0
183	0	0	0	0
184	0	0	0	0
185	0	0	0	0
186	0	0	0	0
187	0	0	0	0
188	0	0	0	0
189	0	0	0	0
190	0	0	0	0
191	0	0	0	0
192	0	0	0	0
193	0	0	0	0
194	0	0	0	0
195	0	1.89%	0	0
196	0	0	0	0
197	0	0	0	0
198	0	0	0	0
199	0	0	0	0
200	0	0	0	0
201	0	0	0	0
202	0	0	0	0
203	0	0	0	0
204	0	0	0	0
205	0	0	0	0
206	0	0	0	0
207	0	0	0	0
208	0	0	0	0
209	0	0	0	0
210	0	0	0	0
211	0	0	0	0
212	0	0	0	0
213	0	0	0	0

214	0	0	0	0
215	0	0	0	0
216	0	0	0	0
217	0	0	0	0
218	0	0	0	0
219	0	0	0	0
220	0	1.92%	0	0
221	0	0	0	0
222	0	0	0	0
223	0	0	0	0
224	0	0	0	0
225	0	0	0	0
226	0	0	0	0
227	0	0	0	0
228	0	0	0	0
229	0	0	0	0
230	0	0	0	0
231	0	0	0	0
232	0	0	0	0
233	0	0	0	0
234	0	1.96%	0	0
235	0	3.92%	2.63%	0
236	0	0	0	0
237	0	0	0	0
238	0	0	0	0
239	0	0	0	0
240	0	0	0	0
241	0	0	0	0
242	0	0	0	0
243	0	0	0	0
244	0	0	0	0
245	0	0	0	0
246	0	0	0	0
247	0	0	0	0
248	0	0	0	0
249	0	0	0	0
250	0	0	0	0
251	0	0	0	0
252	0	0	0	0
253	0	0	0	0
254	0	0	0	0
255	0	0	0	0
256	0	0	0	0
257	0	0	0	0
258	0	0	0	0
259	0	0	0	0
260	0	0	0	0
261	0	0	0	0
262	0	0	0	0
263	0	0	0	0
264	0	0	0	0
265	0	0	0	0
266	0	0	0	0
267	0	0	0	0
268	0	0	0	0
269	0	0	0	0
270	0	0	0	0
271	0	0	0	0
272	0	0	0	0
273	0	0	0	0
274	0	0	0	0
275	0	0	0	0
276	0	0	0	0
277	0	0	0	0
278	0	0	0	0
279	0	0	0	0
280	0	0	0	0

281	0	0	0	0
282	0	0	0	0
283	0	0	0	0
284	0	0	0	0
285	0	0	0	0
286	0	0	0	0
287	0	0	0	0
288	0	0	0	0
289	0	0	0	0
290	0	0	0	0
291	0	0	0	0
292	0	0	0	0
293	0	0	0	0
294	0	0	0	0
295	0	0	0	0
296	0	0	0	0
297	0	0	0	0
298	0	0	0	0
299	0	0	0	0
300	0	0	0	0
301	0	0	0	0
302	0	0	0	0
303	0	0	0	0
304	0	0	0	0
305	0	0	0	0
306	0	0	0	0
307	0	0	0	0
308	0	0	0	0
309	0	0	0	0
310	0	0	0	0
311	0	0	0	0
312	0	0	0	0
313	0	0	0	0
314	0	0	0	0
315	0	0	0	0
316	0	0	0	0
317	0	0	0	0
318	0	0	0	0
319	0	0	0	0
320	0	0	0	0
321	0	0	0	0
322	0	0	0	0
323	0	0	0	0
324	0	0	0	0
325	0	0	0	0
326	0	0	0	0
327	0	0	0	0
328	0	0	0	0
329	0	0	0	0
330	0	0	0	0
331	0	0	0	0
332	0	0	0	0
333	0	0	0	0
334	0	0	0	0
335	0	0	0	0
336	0	0	0	0
337	0	0	0	0
338	0	0	0	0
339	0	0	0	0
340	0	0	0	0
341	0	0	0	0
342	0	0	0	0
343	0	0	0	0
344	0	0	0	0
345	0	0	0	0
346	0	0	0	0
347	0	0	0	0

348	0	0	0	0
349	0	0	0	0
350	0	0	0	0
351	0	0	0	0
352	0	0	0	0
353	0	0	0	0
354	0	0	0	0
355	0	0	0	0
356	0	0	0	0
357	0	0	0	0
358	0	0	0	0
359	0	0	0	0
360	0	0	0	0
361	0	0	0	0
362	0	0	0	0
363	0	0	0	0
364	0	0	0	0
365	0	0	0	0
366	0	0	0	0
367	0	0	0	0
368	0	0	0	0
369	0	0	0	0
370	0	0	0	0
371	0	0	0	0
372	0	0	0	0
373	0	0	0	0
374	0	0	0	0
375	0	0	0	0
376	0	0	0	0
377	0	0	0	0
378	0	0	0	0
379	0	0	0	0
380	0	0	0	0
381	0	0	0	0
382	0	0	0	0
383	0	0	0	0
384	0	0	0	0
385	0	0	0	0
386	0	5.88%	5.88%	0
387	0	0	0	0
388	0	0	0	0
389	0	0	0	0
390	0	0	0	0
391	0	0	0	0
392	0	0	0	0
393	0	0	0	0
394	0	0	0	0
395	0	0	0	0
396	0	0	0	0
397	0	0	0	0
398	0	0	0	0
399	0	0	0	0
400	0	0	0	0
401	0	0	0	0
402	0	0	0	0
403	0	0	0	0
404	0	0	0	0
405	0	0	0	0
406	0	0	0	0
407	0	0	0	0
408	0	0	0	0

Supplemental Figure 7: Effect of ADAR1-3 on tau circRNAs

The wild-type 12->10 and 12->7 expression constructs were co-transfected with the ADAR constructs indicated or GFP as control into HEK293T cells. RNA was analyzed by RNAseq and the editing sites determined as changes A>G.

A. per cent editing at various positions in exons 10 to 12.

B. per cent editing in exons 7 to 9 for the tau circ12->7 RNA.

C. The exon junction reads of tau 12->7 circRNA and tau 12->10 circRNA were determined and normalized for the circHIKP3 junction reads.

A. Tau 12->7 circRNA expression normalized to circHIPK3

circRNA	GFP	ADAR1	ADAR2	ADAR3
HIPK3 (raw expression)	272	443	321	211
MAPT (raw expression)	89	575	372	558
MAPT (normalized expression)	0.33	1.30	1.16	2.64

B. Expression of Tau 12->10 circRNA flanked by tau introns normalized to circHIPK3

circRNA	GFP	ADAR1	ADAR2	ADAR3
HIPK3 (raw expression)	120	127	123	106
MAPT (raw expression)	40	261	169	205
MAPT (normalized expression)	0.33	2.06	1.37	1.93

C. Expression of Tau 12->10 circRNA flanked by ZKSCAN1 introns normalized to circHIPK3

circRNA	GFP[1]	ADAR1	ADAR2	ADAR3
HIPK3 (raw expression)	223	214	174	154
MAPT (raw expression)	4547	12152	3321	1567
MAPT (normalized expression)	20.39	56.79	19.09	10.18

Supplemental Figure 8

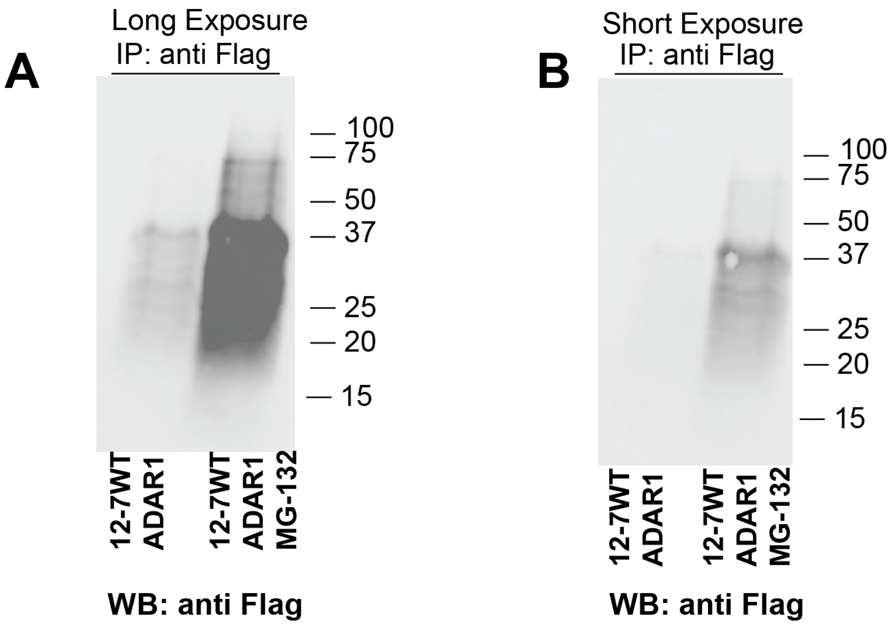

Supplemental Figure 8
The tau 12->7 circRNA expression construct was transfected into HEK293T cells and ubiquitinoylation was blocked by 10 µM MG132 for eight hours prior to cell lysis. The protein was detected with anti flag. **A.** The longer exposure shows an increase of the protein due to MG132 treatment. However, the smearing between the bands does not disappear **B.** (shorter exposure).
